# Identification of *Klebsiella* capsule synthesis loci from whole genome data

**DOI:** 10.1099/mgen.0.000102

**Published:** 2016-12-12

**Authors:** Kelly L. Wyres, Ryan R. Wick, Claire Gorrie, Adam Jenney, Rainer Follador, Nicholas R. Thomson, Kathryn E. Holt

**Affiliations:** ^1^​ Centre for Systems Genomics, University of Melbourne, Parkville, Australia; ^2^​ Department of Biochemistry and Molecular Biology, Bio21 Molecular Science and Biotechnology Institute, University of Melbourne, Parkville, Australia; ^3^​ Infectious Diseases and Microbiology Unit, The Alfred Hospital, Melbourne, Australia; ^4^​ LimmaTech Biologics AG, Schlieren, Switzerland; ^5^​ The Wellcome Trust Sanger Institute, Hinxton, Cambridge, UK; ^6^​ London School of Hygiene and Tropical Medicine, Keppel Street, London, UK

**Keywords:** *Klebsiella* capsule K-locus genomic surveillance

## Abstract

*Klebsiella pneumoniae* is a growing cause of healthcare-associated infections for which multi-drug resistance is a concern. Its polysaccharide capsule is a major virulence determinant and epidemiological marker. However, little is known about capsule epidemiology since serological typing is not widely accessible and many isolates are serologically non-typeable. Molecular typing techniques provide useful insights, but existing methods fail to take full advantage of the information in whole genome sequences. We investigated the diversity of the capsule synthesis loci (K-loci) among 2503 *K. pneumoniae* genomes. We incorporated analyses of full-length K-locus nucleotide sequences and also clustered protein-encoding sequences to identify, annotate and compare K-locus structures. We propose a standardized nomenclature for K-loci and present a curated reference database. A total of 134 distinct K-loci were identified, including 31 novel types. Comparative analyses indicated 508 unique protein-encoding gene clusters that appear to reassort via homologous recombination. Extensive intra- and inter-locus nucleotide diversity was detected among the *wzi* and *wzc* genes, indicating that current molecular typing schemes based on these genes are inadequate. As a solution, we introduce *Kaptive*, a novel software tool that automates the process of identifying K-loci based on full locus information extracted from whole genome sequences (https://github.com/katholt/Kaptive). This work highlights the extensive diversity of *Klebsiella* K-loci and the proteins that they encode. The nomenclature, reference database and novel typing method presented here will become essential resources for genomic surveillance and epidemiological investigations of this pathogen.

## Data Summary

1. Genome data generated and/or analysed in this work are available in the European Nucleotide Archive and/or PATRIC genome database, individual accession numbers are listed in Table S1.

2. Novel K-locus nucleotide sequences have been deposited in GenBank: accession numbers LT603702–LT603735 (https://www.ncbi.nlm.nih.gov/).

3. The *Kaptive* source code, along with the curated K-locus nucleotide and annotation databases have been deposited in GitHub – doi: 10.5281/zenodo.55773 (https://github.com/katholt/Kaptive).

## Impact Statement

*Klebsiella pneumoniae* is a major cause of healthcare-associated infections and an urgent public-health threat for which robust epidemiological surveillance is paramount. These bacteria produce polysaccharide capsules that are important virulence factors, as well as epidemiological markers. Seventy-seven distinct capsule types (K-types) were defined by phenotypic studies in the 1950s–1970s, but the true extent of capsule diversity remains unknown. The increasing availability of whole genome sequences provides an unprecedented opportunity to explore capsule diversity, and here we report our study of the capsule synthesis loci (K-loci) in >2500 *Klebsiella* genomes. We identify a total of 134 distinct K-loci and show that they are extremely diverse, suggesting they encode at least 134 distinct K-types and are subject to unknown, diversifying selective pressures. Furthermore, we present a curated reference database and a new tool for the identification of K-loci from genome sequences, which will greatly assist epidemiological surveillance for *K. pneumoniae*, and other bacterial pathogens for which capsule epidemiology has been shown to be important.

## Introduction

*Klebsiella pneumoniae* and its close relatives, *Klebsiella variicola* and *Klebsiella quasipneumoniae*, are opportunistic pathogens recognized as a significant threat to global health. Antimicrobial resistance, particularly multi-drug resistance and resistance to the carbapenems, is a major concern. Notably, there are a number of globally distributed, multi-drug resistant clones that cause outbreaks of healthcare-associated infections (Munoz-Price *et al.*, 2013; The *et al.*, 2015).

In order to control the emerging threat of *K. pneumoniae sensu stricto, K. variicola* and *K. quasipneumoniae* (hereafter collectively called *Kp*), there is an urgent requirement for genome-based surveillance. Recent advances in understanding population structure (Bialek-Davenet *et al.*, 2014; Holt *et al.*, 2015) highlight immense genomic diversity and provide a framework for tracking this pathogen. Useful strategies involve analyses of lineages or multi-locus sequence types in combination with resistance and virulence gene characterization (Bialek-Davenet *et al.*, 2014), or phylogenetic analysis for outbreak investigation (Snitkin *et al.*, 2012; The *et al.*, 2015). However, reliable methods for tracking *Kp* capsular variation are currently lacking.

The polysaccharide capsule is the outermost layer of the *Kp* cell, protecting the bacterium from desiccation, phage and protist predation (March *et al.*, 2013; Whitfield, 2006). The capsule is also a key virulence determinant. In contrast to capsulated strains, isogenic non-capsulated strains are unable to cause disease in murine infection models (Cortés *et al.*, 2002; Lawlor *et al.*, 2005). In addition, the capsule has been shown to suppress the host inflammatory response (Yoshida *et al.*, 2000), provide resistance to antimicrobial immune-peptides (Campos *et al.*, 2004), complement-mediated killing (Merino *et al.*, 1992) and phagocytosis (Evrard *et al.*, 2010; Lee *et al.*, 2014; March *et al.*, 2013). There are 77 immunologically distinct *Klebsiella* capsule types (K-types) defined by serology (Edmunds, 1954; Edwards & Fife, 1952; Ørskov & Fife-Asbury, 1977). However, serological typing requires specialist techniques and reagents not available to most microbiology laboratories, so is very rarely applied. Furthermore, 10–70 % of *Kp* isolates are serologically non-typeable, because either they express a novel capsule (common for clinical isolates) or they are non-capsulated (Cryz *et al.*, 1986; Jenney *et al.*, 2006; Tsay *et al.*, 2002).

*Kp* employ a Wzy-dependent capsule synthesis process (Rahn *et al.*, 1999; Whitfield, 2006) for which the associated genes are in the capsule synthesis locus (K-locus), which is 10–30 kbp in length (Arakawa *et al.*, 1995; Chuang *et al.*, 2006; Fevre *et al.*, 2011; Pan *et al.*, 2008, 2015; Shu *et al.*, 2009). The K-locus includes a set of common genes in the terminal regions that encode the core capsule biosynthesis machinery (e.g. *galF*, *wzi*, *wza*, *wzb*, *wzc*, *gnd* and *ugd*). The central region is highly variable, encoding the capsule-specific sugar synthesis, processing and export proteins, plus the core assembly components Wzx (flippase) and Wzy (capsule repeat unit polymerase) (Pan *et al.*, 2015).

K-locus nucleotide sequences and annotations are now available for a large number of *Kp* isolates, including the 77 K-type reference strains (Chuang *et al.*, 2006; Deleo *et al.*, 2014; Follador *et al.*, 2016; Pan *et al.*, 2008, 2015; Shu *et al.*, 2009; The *et al.*, 2015; Wyres *et al.*, 2015). Serological K-types are generally defined by distinct sets of protein-encoding genes in the variable central region of the K-locus; however, two types (K22 and K37) are distinguished by a point mutation resulting in a premature stop codon that affects acetyltransferase function (Pan *et al.*, 2015).

A number of molecular K-typing schemes have been developed, including RFLP (‘C-typing’) (Brisse *et al.*, 2004), *wzi* and *wzc* typing (Brisse *et al.*, 2013; Pan *et al.*, 2013), and capsule-specific *wzy* PCR-based typing (Pan *et al.*, 2008; Yu *et al.*, 2007). These methods are less technically challenging and more discriminatory than serological techniques (Brisse *et al.*, 2004, 2013; Pan *et al.*, 2013). None have been widely adopted, although the single gene *wzi* and *wzc* typing schemes are gaining traction in the high-throughput sequencing era (Bialek-Davenet *et al.*, 2014; Bowers *et al.*, 2016; Zhou *et al.*, 2016). Within the *wzi* scheme, unique alleles are associated with specific K-types (Brisse *et al.*, 2013). Within the *wzc* scheme, K-types are assigned based on the level of *wzc* nucleotide similarity to a reference sequence, with a threshold of 94 % (Pan *et al.*, 2013). Regardless of the method, a substantial proportion of isolates remain non-typeable; consequently, the true extent of *Kp* capsule diversity remains unknown.

Here, we report the K-loci from a collection of 2503 *Kp*. We have identified 31 novel K-loci, and have provided evidence that limited diversity remains to be discovered. We have defined a standardized nomenclature, provided a curated K-locus reference database and introduced *Kaptive*, a tool for rapid identification of reference K-loci from genome data, which will greatly facilitate surveillance efforts and evolutionary investigations of this important pathogen.

## Methods

### Sequences.

We obtained a total of 2600 *Kp* genomes (2021 publicly available and 579 novel genomes from a diverse set collected in Australia). Sequence reads were generated locally or obtained from the European Nucleotide Archive (accession numbers are listed in Table S1, available with the online Supplementary Material); 916 genomes that were publicly available as assembled contigs only were downloaded from PATRIC (Wattam *et al.*, 2014) and the NCTC3000 Project (Wellcome Trust Sanger Institute – http://www.sanger.ac.uk/resources/downloads/bacteria/nctc/). For isolates sequenced in this study (*n*=579), DNA was extracted and libraries prepared using the Nextera XT 96 barcode DNA kit and 125 bp paired-end reads were generated on the Illumina HiSeq 2500 platform.

All paired-end read sets were filtered for a mean Phred quality score ≥30, then assembled *de novo* using SPAdes v3 (Bankevich *et al.*, 2012). Genomes were excluded from the study if they were duplicate samples, or if there was evidence of contamination or mixed culture measured by: (i) <50 % reads mapping to the NTUH-K2044 reference chromosome (accession number: AP006725.1); (ii) the ratio of heterozygous/homozygous single nucleotide polymorphism (SNP)calls compared to the reference chromosome exceeding 20 %; (iii) the total assembly length being >6.5 Mb, or >6.0 Mb with evidence of >1 % non-*Klebsiella* read contamination as determined by MetaPhlAn (Segata *et al.*, 2012); or (v) the assembly being low quality, i.e. total length <5 Mb.

### Existing high-quality K-locus reference sequences.

K-locus nucleotide sequences for each of the 77 K-type references and 2 serologically non-typeable strains published elsewhere (Arakawa *et al.*, 1995; Chuang *et al.*, 2006; Fevre *et al.*, 2011; Pan *et al.*, 2008, 2015; Shu *et al.*, 2009) were obtained from GenBank or directly from the authors (accession numbers are shown in Table S2). A total of 12 additional K-locus sequences had been published prior to the K-type references (Chen *et al.*, 2014; Deleo *et al.*, 2014; The *et al.*, 2015; Wyres *et al.*, 2015); we have compared these loci to those of the 77 K-type references (Arakawa *et al.*, 1995; Chuang *et al.*, 2006; Fevre *et al.*, 2011; Pan *et al.*, 2008, 2015; Shu *et al.*, 2009) and identified 7 that were novel. These 7 novel loci plus 17 distinct loci described in our recent survey (Follador *et al.*, 2016) were added to the non-redundant list of K-locus reference sequences, resulting in a total of 103 loci (see Table S2).

### Identification of novel K-loci.

In order to identify novel K-loci, we first classified each genome by similarity to previously known loci. blastn (Camacho *et al.*, 2009) was used to search each genome assembly for sequences with similarity to those of annotated K-locus coding sequences (CDSs) usually located between *galF* and *ugd* inclusive (minimum coverage 80 %, minimum identity 50 %). Transposase CDSs present in the published K-locus reference sequences were excluded from this analysis since they are not K-locus specific. Up to three missing CDSs were tolerated for K-locus assignment, to allow for assembly problems and insertion sequence (IS) insertions (see the Supplementary Methods and Fig. S1). This approach successfully distinguished the 77 K-type reference loci (with the exception of K22 and K37).

Genomes that could not be assigned a K-locus were investigated further: blastn was used to identify the *galF* and *ugd* genes within the assembly, and single contig loci were extracted. The assembly graph viewer Bandage (Wick *et al.*, 2015) was used to identify K-loci that did not assemble on a single contig or where *galF* and/or *ugd* where missing. Loci were clustered, with identity and coverage thresholds of 90 %, using cd-hit-est (Fu *et al.*, 2012; Li & Godzik, 2006). A representative sequence from each cluster was annotated with prokka (Seemann, 2014), using all proteins in the 77 reference K-type loci as the primary annotation database. Novel K-locus sequences were deposited in GenBank (accession numbers LT603702–LT603735; also included in the *Kaptive* database at https://github.com/katholt/Kaptive/).

Recombination in K-loci was investigated by aligning nucleotide sequences for the eight common genes (extracted from the reference annotations) using muscle (Edgar, 2004). This generated a 9944 bp concatenated gene alignment that was used as input for maximum likelihood (ML) phylogenetic inference with RAxML (Stamatakis, 2006) (best scoring tree from five runs each of 1000 bootstrap replicates with gamma model of rate heterogeneity), and recombination analysis using ClonalFrameML (Didelot & Wilson, 2015) (run for 1000 simulations and using the ML phylogeny as the starting tree).

### Amino acid clustering.

Predicted amino acid sequences of all annotated K-locus coding regions were translated from the DNA sequences using BioPython and clustered with cd-hit (Fu *et al.*, 2012; Li & Godzik, 2006) (90, 80, 70, 60, 50, 40 % identity). We explored the co-occurrence of predicted protein clusters present in three or more K-loci each (excluding the common proteins and the initiating glycosyltransferases, WbaP and WcaJ, *n*=115 clusters for analysis): pairwise Jaccard similarity scores were calculated as J (A, B)=A∩B/A∪B and were used to draw a weighted edge graph with the igraph R package v 1.0.1 (Csardi & Nepusz, 2006). A weight threshold was determined empirically as 0.61 and all edges for which J<0.61 were removed.

### *wzc* and *wzi* nucleotide sequence determination.

We used srst2 (Inouye *et al.*, 2014) to determine *wzi* alleles defined in the *Kp* BIGSdb (Institut Pasteur *Klebsiella pneumoniae* BIGSdb). blastn was used to determine alleles in genomes available only as assemblies. Novel alleles were submitted to the *Kp* BIGSdb for official designation. *wzc* sequences were extracted from genome assemblies by blastn search against a database of published alleles (Pan *et al.*, 2013). Sequences were aligned with muscle (Edgar, 2004) and pairwise nucleotide divergences calculated.

### *Kaptive*, a tool for identification of K-loci in genome data.

We developed an extended procedure for identification and assessment of full-length K-loci among bacterial genomes based on blast analysis of assemblies. The procedure has been automated and is implemented in a freely available open source software tool, *Kaptive* (https://github.com/katholt/Kaptive). For full details see Supplementary Methods and Results.

### Comparison of *Kaptive*, *wzi* and *wzc* typing results.

*Kaptive, wzi* and *wzc* typing were applied to 86 genomes that had matched serological typing information available (Table S3). *wzi* alleles were determined as described above, and used to predict serotypes by comparison to the *Kp* BIGSdb. *wzc* sequences were extracted as above and genomes were assigned to serotypes if the sequence was <6 % divergent from the corresponding reference (Pan *et al.*, 2013).

## Results

### Identification of novel K-loci

We investigated 2503 genomes that passed our quality-control standards (see Methods): 2298 *K. pneumoniae sensu stricto*, 144 *K. variicola*, 57 *K. quasipneumoniae* and 4 unclassified *Klebsiella* spp. (Tables 1 and S1). Also included were 10 publicly available genomes representing the more distantly related *Klebsiella oxytoca* (Table S4), as we hypothesized that they may share K-loci with *Kp*. Isolates had been collected between 1932 and 2014 (Fig. S2a), and from eight geographical regions spanning six continents (Fig. S2b).

**Table 1.** *Kp* genomes investigated in this study

Source	Count	Reference	Note
Bialek-Davenet *et al.*	33	Bialek-Davenet *et al.* (2014)	Investigation of multi-drug resistant and hypervirulent clones
Bowers *et al.*	160	Bowers *et al.* (2015)	Isolates mostly of clonal group 258
Davis *et al.*	77	Davis *et al.* (2015)	Isolates from human urinary tract infections and animal meats in Arizona, USA
Deleo *et al.*	69	Deleo *et al.* (2014)	Isolates of clonal group 258
Ellington, M	185	Unpublished	Multi-drug resistant isolates from a hospital in Cambridge, UK
Holt *et al.*	274	Holt *et al.* (2015)	Global diversity study
Lee *et al.*	27	Lee *et al.* (2016)	Isolates from pyogenic liver abscess disease, Singapore
NCTC3000	81	Wellcome Trust Sanger Centre website	Isolates from the Public Health England NCTC reference collection
PATRIC	811	Wattam *et al.* (2014)	Genome assemblies submitted to GenBank
Stoesser *et al.*	69	Stoesser *et al.* (2013)	Isolates from health-care associated infections, Oxford, UK
Stoesser *et al.*	55	Stoesser *et al.* (2014)	Isolates collected during an outbreak in Nepal
Struve *et al.*	67	Struve *et al.* (2015)	Predominantly isolates of clonal group 23
The *et al.*	76	The *et al.* (2015)	Isolates from two outbreaks in Nepal
Wand *et al.*	35	Wand *et al.* (2015)	Historical isolate collection (Murray collection)
Novel isolates	484	This study	Diverse collection of Australian hospital surveillance isolates

NCTC, National Collection of Type Cultures.

A total of 1371 genomes could be putatively assigned to 63 of the 77 K-type reference loci by the blast screening approach. A further 918 genomes were assigned to 1 of 25 previously published K-loci that are distinct from the K-type reference loci. Among the remaining 213 genomes, 106 were assigned to 29 novel K-loci, bringing the total to 132 (Table S1). Nine genomes harboured deletion or IS variants of known or novel loci (see below). For 93 genomes (3.7 %), no K-locus could be determined; however, we found three or more K-locus-associated genes in all such genomes and hypothesize the lack of assignment was attributable to low read depth and/or fragmented genome assembly rather than complete K-locus deletion.

The K66 and K74 loci were identified in one and four *K. oxytoca* genomes, respectively. Two novel K-loci were identified from three *K. oxytoca* genomes, increasing the total known K-loci to 134 (Table S2).

**Fig. 1.**
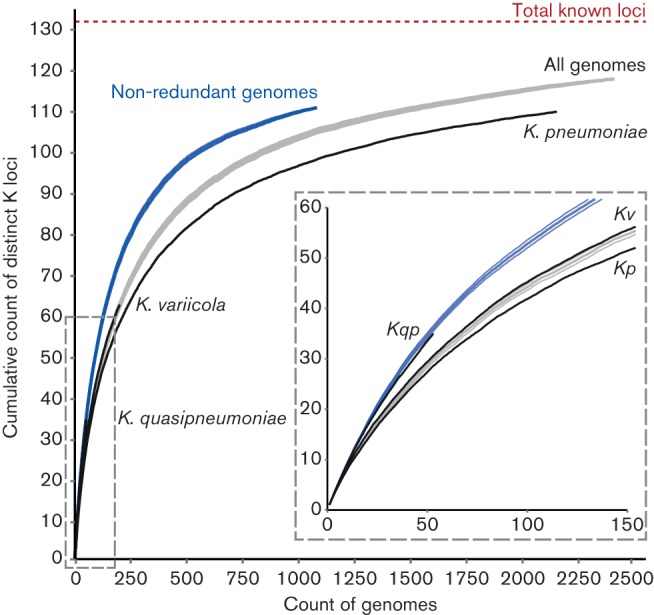
Rate of discovery of distinct K-loci with increasing genome sample size. Curves indicate the accumulation of distinct K-loci (mean±se) in different genome sets: grey, all genomes (*n*=2410, excluding *K. oxytoca*); blue, non-redundant genomes (*n*=1081, excludes genomes from investigations of disease outbreaks and specific clonal groups); black, species-specific genome sets (*K. pneumoniae* refers to *K. pneumoniae sensu stricto*, se not shown). The inset shows a magnified view of the bottom-left section of the plot, as indicated by the dashed box.

We estimated the extent to which we had captured the repertoire of K-locus diversity in the *Kp* population (Fig. 1). Rarefaction curves were estimated from: (i) the full genome set for which K-loci were assigned (*n*=2410); (ii) a ‘non-redundant’ genome set from which highly biased sub-samples such as outbreaks were removed (*n*=1081); and (iii) genomes from the non-redundant set representing each of the distinct species, *K. variicola*, *K. quasipneumoniae* and *K. pneumoniae sensu stricto*. In comparison to that for the full genome set, the non-redundant curve better represents the true diversity of the *Kp* population. Note that neither reached the total number of known K-loci, since 13 of the serologically defined K-loci (Pan *et al.*, 2015) were not represented in our 2503 genomes. The rarefaction curves for each of the three *Klebsiella* species within the non-redundant dataset were similar to one another, indicating comparable levels of capsule diversity within each species (Fig. 1).

### K-locus nomenclature and reference database

We used a standardized K-locus nomenclature based on that proposed for *Acinetobacter baumannii* (Kenyon & Hall, 2013). Each distinct K-locus was designated as KL (K-locus) and a unique number. The K-type reference K-loci were assigned the same number as the corresponding K-type, e.g. K1 is encoded by the KL1 locus. K-loci for which K-types have not yet been phenotypically defined were assigned identifiers starting from 101 (note KL101 and KL102 correspond to loci previously named KN1 and KN2).

K-loci with IS insertions were distinguished from orthologous IS-free variants by using −1,–2. This nomenclature was consistently applied to the 10 K-type reference K-loci published elsewhere that include one or more ISs (Fevre *et al.*, 2011; Pan *et al.*, 2008, 2015; Shu *et al.*, 2009). Deletion variants derived from a known K-locus were given the suffix -D1, -D2, etc. A complete *Klebsiella* K-locus reference database is available at https://github.com/katholt/Kaptive (see Table S2 and Supplementary Results for details).

### K-locus epidemiology

The genome collection studied here does not represent a systematic sample and, thus, is not appropriate for detailed exploration of epidemiological questions. However, it is of interest to note the following three points. (i) Among the non-redundant genome set (*n*=1081), 90 % of K-loci identified were represented by just 67 distinct types, suggesting that many K-loci are rare in the *Kp* population, while others are more common. The five most common K-loci accounted for 20 % of those identified (KL2 5 %, KL1 4 %, KL21 4 %, KL17 4 %, KL30 3 %; see Table S1). (ii) There is evidence that K-types are shared across environmental and source populations. Twenty-eight K-loci were identified among isolates from retail meats (chicken, pork and turkey) for which genomes were originally published by Davis *et al.* (2015), and we found all (100 %) of these K-loci among human infection isolates in our data set. A total of 37 distinct K-loci were identified for 51 isolates from bovine hosts in the *Kp* global diversity study (Holt *et al.*, 2015), and we found 35 (94.6 %) of these among human isolates and 12 (32.4 %) among the retail meat isolates. (iii) Our data set included 644 genomes from the globally distributed, multi-drug resistant clonal group 258, which was previously shown to contain extensive K-locus diversity (Bowers *et al.*, 2015; Wyres *et al.*, 2015). We identified a total of 32 distinct K-loci, plus 1 IS and 1 deletion variant amongst clonal group 258 genomes (Table S1). The most common K-loci were KL106 (*n*=125, 19.4 %) and KL107 (*n*=437, 67.9 %), which match the loci previously cited in the literature in association with *wzi* alleles 29 and 154, respectively (Bowers *et al.*, 2015; Deleo *et al.*, 2014; Wyres *et al.*, 2015).

### K-locus structures

The novel K-loci identified in this study conformed to the common structure described elsewhere (Fig. S3) (Pan *et al.*, 2015; Rahn *et al.*, 1999; Shu *et al.*, 2009). We also identified six deletion variants: KL5-D1, KL20-D1, KL30-D1, KL62-D1, KL106-D1 and KL107-D1. Each of these was missing several common genes, but the remaining regions were homologous to existing K-loci represented in our genome collection. We suggest that the latter K-loci represent the ancestral forms that have subsequently lost one or more regions through deletion events; thus, generating the variants described here. Isolate NCTC10004 (recorded as serotype K11 in the UK National Collection of Type Cultures) and four other genomes carried K-loci that were nearly identical to the previously published K11 reference sequence (Pan *et al.*, 2015). However, the latter lacked the essential *wzx* gene plus two other neighbouring genes, and was not identified among any other genomes. We assume the NCTC10004 locus represents the full length KL11 locus and designate the original K11 reference as KL11-D1 (it is unclear whether the original sequenced reference isolate had retained the ability to produce a capsule, since the serological typing was performed decades earlier) (Pan *et al.*, 2015).

**Fig. 2.**
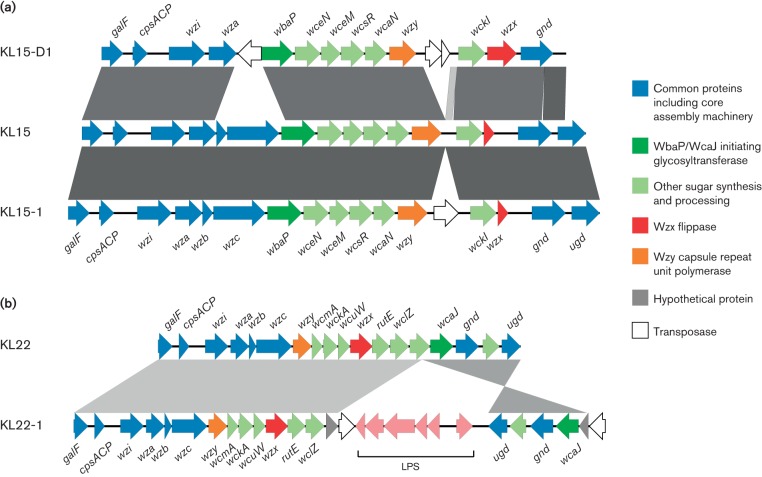
Example K-locus structures and comparisons. CDSs are represented as arrows coloured by predicted function of the protein product and labelled with gene names where known. Grey bars indicate regions of similarity identified by blast comparison, darker shading indicates higher sequence identity. (a) Comparison of deletion variant KL15-D1 and IS variant KL15-1 with the synthetic KL15 locus. (b) Comparison of the IS variant KL22-1 with the K-type reference KL22 locus. The downstream LPS (LPS synthesis) operon (pink arrows) has been translocated into the K-locus.

In four of the deletion variants, the deleted region was replaced by an IS, which may have mediated the deletion. Of the other IS-related variants, KL157-1 contained an IS*903* family IS without an obvious deletion. In addition, we identified two novel IS variants of K-type reference loci (KL15-1 and KL22-1) and five IS-free variants of K-type reference loci (KL3, KL6, KL38, KL57 and KL81), plus one other previously published K-locus (KL103). The KL22-1 locus included a translocation of part of the nearby lipopolysaccharide (LPS) locus to the centre of the K-locus, plus an inversion of the 3′ K-locus region (Fig. 2). The translocated and inverted regions were bound at each end by a copy of IS*Kpn26*.

**Fig. 3.**
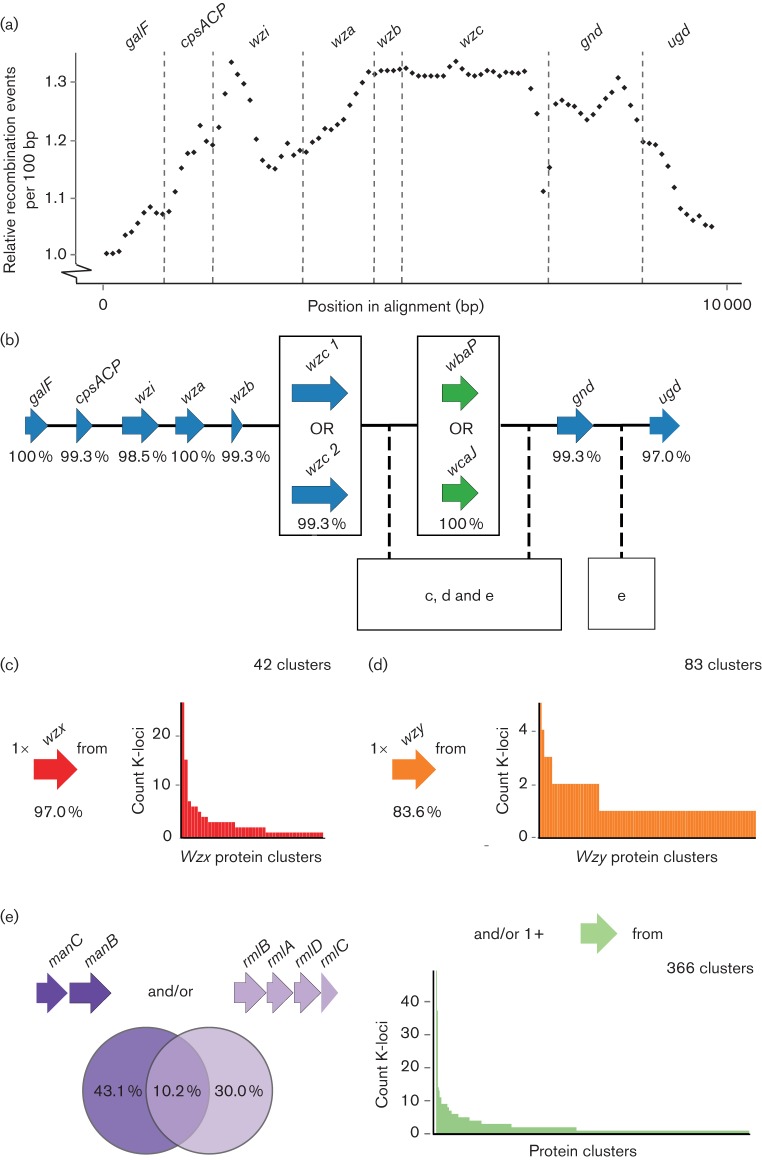
Composition and diversity of *Klebsiella* K-loci. (a) Putative recombination events among the common K-locus genes. Values plotted are the relative number of events per 100 bp window, inferred using ClonalFrameML. (b) Representation of a generalized K-locus structure. Arrows represent K-locus coding regions coloured by predicted protein product as in Fig. 2. Percentage values indicate the number of reference K-loci containing each gene (total 134 references). Note that 13 of the K-locus references partially or completely exclude *ugd*, although it is known to be present in 11 of these loci (Pan *et al.*, 2015). Thus, these 11 were counted as containing *ugd*. (c, d, e) Diversity of proteins encoded by *wzx* (c), *wzy* (d) and sugar processing genes (e) annotated amongst the 134 K-locus reference sequences. The locations within this structure at which *wzx* (c), *wzy* (d) and sugar processing genes (e) have been found to occur are indicated. Bar charts indicate the frequency of each predicted protein cluster.

We used ClonalFrameML (Didelot & Wilson, 2015) to identify putative recombination events within the common K-locus genes. Analysis of the nucleotide sequences from each of the 134 reference K-loci identified a high number of such events (*n*=382), which were not distributed equally across the nucleotide alignment (Fig. 3a); rather the genes closest to the central variable region were affected by a greater number of recombination events compared to those at the ends of the locus.

### Variation in K-locus gene content

A total of 2675 predicted proteins from 134 complete K-loci were clustered using cd-hit (Fu *et al.*, 2012; Li & Godzik, 2006). As the identity threshold was reduced, the number of clusters continued to fall (from 1496 at 90 % identity to 508 at 40 % identity) and showed no signs of stabilizing (Fig. S4). At 40 % identity, which we believe is the lower bound for sensible comparison, the core capsule assembly proteins GalF, Wzi, Wza, Wzb, Gnd and Ugd each formed a single cluster and were present in nearly all loci (Fig. 3b). The Wzc sequences clustered into two groups and each locus encoded one Wzc protein (except KL50). In contrast, Wzx (flippase) clustered into 42 groups and Wzy (capsule repeat unit polymerase) clustered into 83 groups, highlighting the extreme diversity of these proteins compared to other core capsule assembly machinery proteins (Fig. 3c, d).

There were 374 clusters among the remaining proteins, most of which were associated with sugar synthesis and processing (Fig. 3). The initiating sugar transferase proteins, WbaP and WcaJ, were grouped into two clusters. These proteins are considered essential for capsule synthesis. Concordantly, each locus encoded a single protein from one of these two clusters. RmlB, RmlA, RmlD and RmlC, which are associated with rhamnose and typically encoded together in a single operon, were each represented by a single cluster. Similarly, the mannose synthesis and processing proteins, ManC and ManB, were grouped into a single cluster each. The associated operons *rmlBADC* and *manCB* were present in 55 and 73 K-loci, respectively (14 contained both operons; Fig. 3). In contrast, 360 of the remaining 366 protein clusters were present in fewer than ten K-loci each (Fig. 3e).

**Fig. 4.**
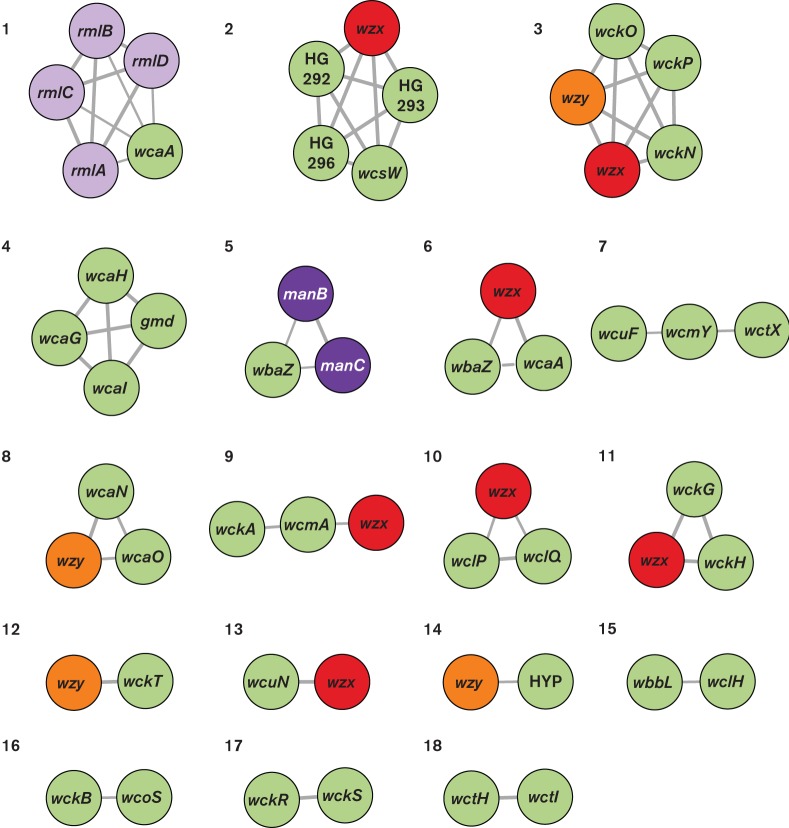
Co-occurrence of *wzx, wzy* and sugar processing genes across reference K-loci. Nodes represent genes, labelled by name and coloured by protein product as in Figs 2 and 3: Wzx (red), Wzy (orange), mannose synthesis/processing proteins (dark purple), rhamnose synthesis/processing proteins (light purple), other proteins (green). Edge widths are proportional to Jaccard index (J) and are shown for all pairs where *J*≥0.61. Numbers represent co-occurrence group assignments as defined in Table S5. HYP, Hypothetical protein.

Co-occurrence analysis identified 18 correlated groups of K-locus proteins, each comprising two to five protein clusters (pairwise Jaccard similarity ≥0.61 for all pairs in the group; Fig. 4, Table S5). One group included the four Rml protein clusters; interestingly, this group also included a WcaA glycosyltransferase, which was present in 67.3 % of *rmlBADC*-containing K-loci and no *rmlBADC*-negative loci (χ^2^=70.09, *P* value <2.2×10^−16^, two-sided proportion test). Similarly, another group included the ManCB proteins and the putative mannosyl transferase, WbaZ, which was present in 65.8 % of *manCB*-containing K-loci and one *manCB*-negative locus (χ^2^=56.159, *P* value=6.683×10^−14^, two-sided proportion test). In addition, several groups included proteins for which the genes were located sequentially in their K-loci (e.g. *wckG*, *wckH* and *wzx* in KL12, KL29 and KL42) consistent with linked gene transfer.

### Diversity of *wzc* and *wzi* gene sequences

**Fig. 5.**
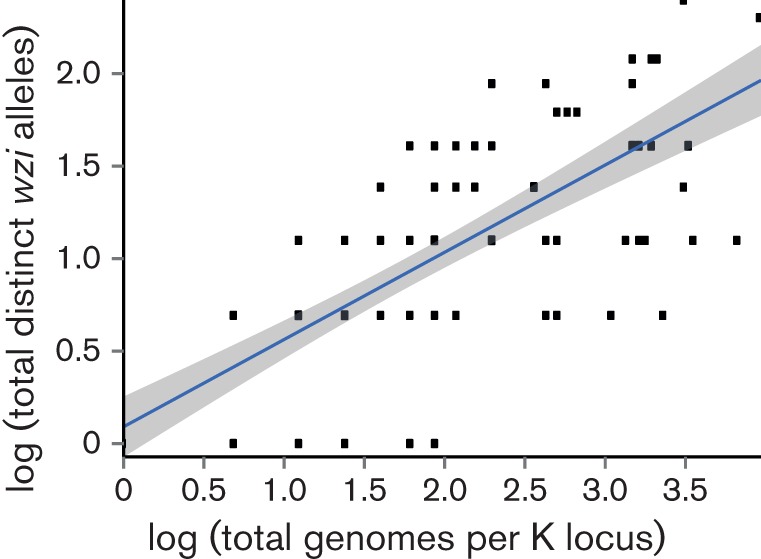
Allelic diversity of *wzi*. Within K-locus *wzi* allelic diversity increases with total K-locus representation. The blue line represents the least-squares regression and grey shading indicates the 95 % confidence interval.

We sought to explore the utility of the existing *wzi* and *wzc* molecular capsule typing methods by characterizing *wzi* and *wzc* nucleotide sequence diversity and their association with K-loci. We confidently assigned *wzi* alleles to 2461 *Kp* genomes, including 390 distinct alleles, 218 of which were novel. Median pairwise nucleotide divergence was 7 %. Among the non-redundant genome set, there were 54 *wzi* alleles represented by at least five genomes, and of these 15 (28 %) were associated with more than one K-locus (Table S1). Among the 67 K-loci for which we had ≥5 representative sequences, 64 (95.5 %) were associated with two or more *wzi* alleles, and there was a general trend towards increasing *wzi* allelic diversity with increasing K-locus representation (Fig. 5).

**Fig. 6.**
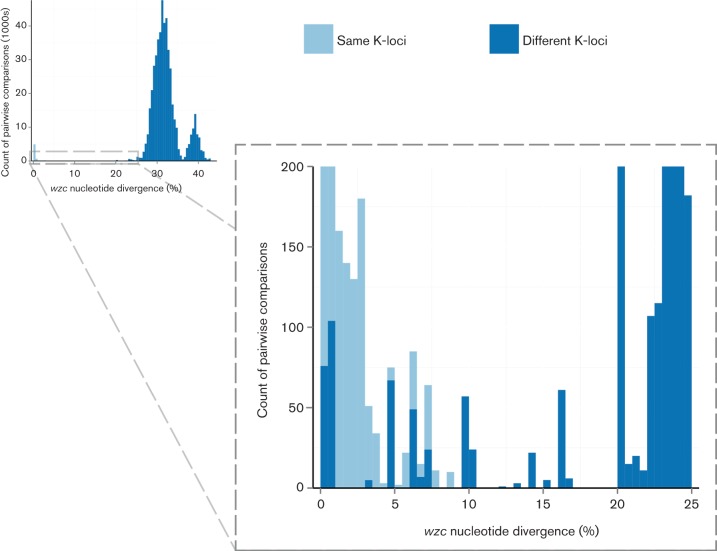
*wzc* nucleotide diversity. Barplots showing distribution of pairwise *wzc* nucleotide divergence for pairs of genomes with the same (light blue) or different (dark blue) K-loci. The inset shows a magnified view of the lower end of the distribution.

We extracted *wzc* sequences from 1041 of 1081 genomes in the non-redundant set (Fig. 6). In general, genomes sharing the same K-locus (6262 pairwise observations) showed lower *wzc* nucleotide divergence than those with different K-loci (491 775 pairwise observations), but the distributions overlapped substantially (Fig. 6). Notably, there were five distinct combinations of K-loci for which one or more pairs harboured *wzc* sequences that were <6 % divergent [the cut-off for K-type assignment as described in Pan *et al.* (2013)]: KL1 and KL112, KL9 and KL45, KL15 and KL52, KL30 and KL104, KL40 and KL135. Conversely, two K-loci (KL45, KL112) had more than 25 % *wzc* nucleotide divergence between representatives of the same K-locus.

### *Kaptive* – capsule locus (K-locus) typing and variant evaluation from genome data

**Fig. 7.**
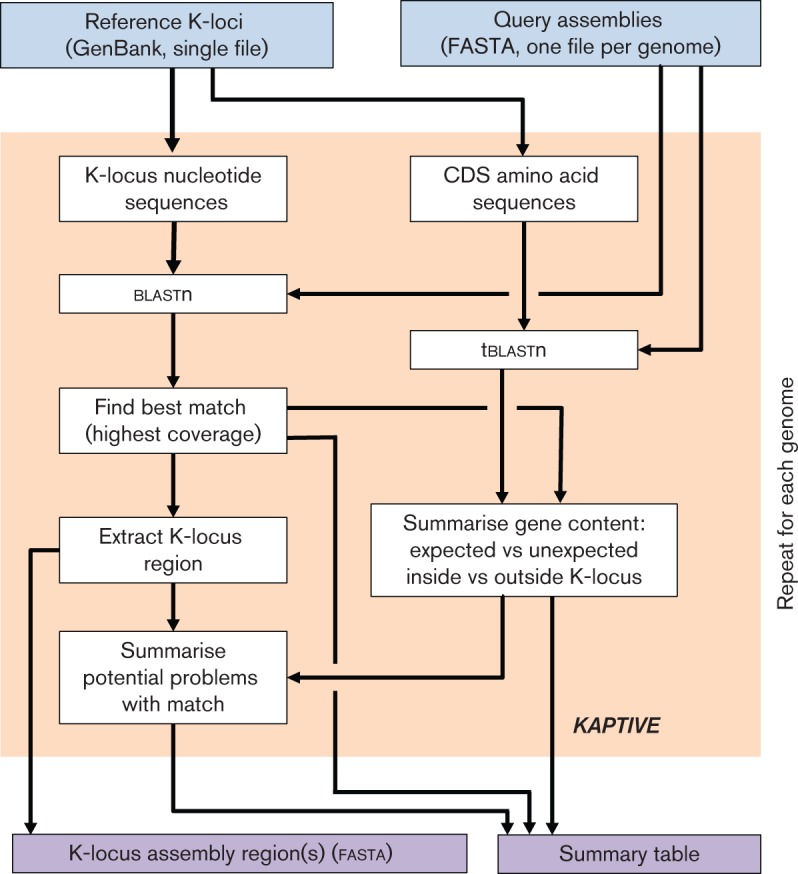
Summary of the *Kaptive* analysis procedure. *Kaptive* takes as input a set of annotated reference K-loci in GenBank format and one or more genome assemblies, each as a single fasta file of contigs. *Kaptive* performs a series of blast searches to identify the best-match K-locus in the query genome and assess the presence of genes annotated in the best-match locus (expected genes) and those annotated in other loci (unexpected genes) both within and outside the putative K-locus region of the query assembly. The output is a fasta file containing the nucleotide sequence(s) of the K-locus region(s) for each query assembly and a table summarizing the best-match locus, gene content and potential problems with the match (e.g. the assembly K-locus region is fragmented, expected genes are missing from the K-locus region or at low identity, or unexpected genes are present) for each query assembly. For the user’s convenience, *Kaptive* will also use blastn to find and report the best match *wzi* and *wzc* allele sequences, as defined in the *Kp* BIGSdb (not shown in schematic).

To facilitate easy identification of K-loci from genome assemblies, we developed the software tool *Kaptive* (see Fig. 7 and Supplementary Methods). We used *Kaptive* with our primary K-locus reference database (excluding IS and deletion variants) to rapidly type the K-loci in our collection of 2503 *Kp* genomes, and obtained confident K-locus calls for 2412 genomes (96.4 %, see Supplementary Results for further details).

We compared the K-locus calls from *Kaptive*, *wzc* and *wzi* typing to serological typing results for 86 isolates for which both genome and serology data were available (Holt *et al.*, 2015; Jenney *et al.*, 2006; NCTC3000 Project; Table S3). Five of six isolates that were non-typeable by serological techniques were identified by *Kaptive* as carrying KL16, KL54, KL81, KL111 and KL149. The KL16, KL54 and KL81 calls were in agreement with *wzc* and *wzi* typing results; the other two K-loci were not present in the *wzi* or *wzc* schemes and so were not typeable by those methods. Among the 80 serologically typeable isolates, the three molecular methods were generally in agreement with one another, although concordance with recorded phenotypes was quite low (65–74 %, Table S3). Call rates were highest for *Kaptive* (95 %), followed by *wzc* (89 %) and *wzi* typing (75 %).

## Discussion

The number of distinct *Klebsiella* K-loci (now 134) is striking and exceeds that described for K-loci in other bacterial species, such as *A. baumannii* and *Streptococcus pneumoniae.* Furthermore, the diversity is an order of magnitude greater than that recently described for *Klebsiella* LPS, the other major *Klebsiella* surface antigen (Follador *et al.*, 2016). This suggests that the K-locus is subject to strong diversifying selection. Given that these bacteria are not obligate pathogens and are ubiquitous in non-host-associated environments (Bagley, 1985; Podschun & Ullmann, 1998), the factors driving selection may not be host immune pressures but may include phage and/or protist predation.

Two novel K-loci, plus the KL66 and KL74 loci, were identified from *K. oxytoca*, a close relative of *Kp*. Little is known about *K. oxytoca* capsules, but one previous report also identified several *Kp*-associated capsules among *K. oxytoca* isolates (Ishihara *et al.*, 2012). These findings indicate that *K. oxytoca* is able to exchange genetic material with *Kp* and, thus, represents a potential reservoir of virulence and other genes.

Our analysis confirms there are strong constraints on the structure of K-loci (Fig. 3). However, our data also reveal the extensive diversity of proteins encoded in the variable central region. The associated genes ranged in frequency from 0.7 to 54.7 % of the K-loci. Among those represented in at least three loci, approximately half co-occurred in groups ranging from two to five genes.

The molecular evolution driving K-locus diversification is not well understood, but likely includes a combination of point mutation, IS-mediated rearrangements and homologous recombination within the locus. In *A. baumannii* (Holt *et al.*, 2016; Schultz *et al.*, 2016) and *S. pneumoniae* (Wyres *et al.*, 2013), it has been shown that recombination within the K-locus can drive capsule exchange between distinct clones. We recently speculated that this may also be true for *Kp* (Wyres *et al.*, 2015) and the recombination analysis presented here supports this theory. The genes closest to the central variable region of the K-locus (i.e. *wzb, wzc* and *gnd*) showed evidence of the greatest number of recombination events, consistent with the hypothesis that they act as regions of homology for recombination events that shuffle the central region of the locus.

Prediction of capsule phenotypes from genome data is complex, as capsule expression is highly regulated and involves genes outside the K-locus region (Hsu *et al.*, 2011). However, it is likely that K-loci encoding distinct sets of proteins are associated with distinct phenotypes, as is the case for the vast majority of K-type reference strains (Pan *et al.*, 2015). Therefore, our data suggest that there are at least 134 distinct *Klebsiella* K-types. This is a lower bound estimate, particularly because our analysis did not capture differences that may arise from point mutations and small-scale insertions or deletions [e.g. in the case of K22 and K37, described by Pan *et al.* (2015)]. Furthermore, while we did not attempt to thoroughly characterize IS variants, several were apparent. The potential functional impacts of IS insertions likely depend on their location in the locus, but may include up-regulation, loss of capsule production or more subtle changes in sugar structures (Bentley *et al.*, 2006; Hsu *et al.*, 2016; Salter *et al.*, 2012; Uria *et al.*, 2008).

Serological typing of *Klebsiella* isolates is notoriously difficult and rarely performed. We were able to compare genotypes (whole-locus typing using *Kaptive*, as well as *wzi* and *wzc* typing schemes) with phenotypes for just 86 isolates for which both sequences and serotypes were available. Of the 19 discordant genotype versus phenotype results, 2 were due to deletion variants and were resolved by running *Kaptive* with the K-locus variants database. Interestingly, one of these isolates was non-typeable by serology, *wzi* or *wzc* typing, but recognized as a specific K-locus deletion variant by *Kaptive*. This highlights a benefit of our whole-locus typing approach; it provides epidemiologically relevant information even when the K-locus is interrupted. Another isolate was serologically typed as K54 but genotyped by *Kaptive* as KL113, which has sequence homology with KL54 (>84 % nucleotide identity over 76 % of the locus) and may encode a serologically similar or cross-reacting capsule. The other cases of discordance had no obvious explanation and may result from serological typing errors or from mutations arising during subculture (as identified for the K11 reference isolate above), neither of which we were able to check. Some discordance may also be due to unpredictable serological cross-reactions.

Given the problems with serotyping and the comparative robustness and widespread access to genome sequencing, we anticipate that genotyping will remain the preferred method for tracking capsular diversity in *Klebsiella*. Due to the extensive diversity and potential for ongoing evolution, we strongly advocate for classification based on complete, or near complete K-locus sequences, rather than single genes such as *wzi* or *wzc,* which can be misleading due to substitutions and horizontal gene transfer such as that described in this work. *Kaptive* analyses the full-length K-locus nucleotide sequence and assesses the presence of all K-locus-associated genes by protein blast search; thus, the approach is resilient to spurious results that may arise due to sequence divergence. Furthermore, the information provided allows users to determine confidence in the results and to identify putative novel K-loci or variants of known loci if desired. Along with the curated reference databases, this new tool will greatly facilitate evolutionary investigations and genomic surveillance efforts for *Kp* and other bacterial pathogens.

## Supplementary Data

626 Supplementary File 1

## Supplementary Data

900 Supplementary File 2

## Supplementary Data

61 Supplementary File 3

## Supplementary Data

101 Supplementary File 4

## Supplementary Data

147 Supplementary File 5
