# Supplementary material for: Identification of Klebsiella capsule synthesis loci from whole genome data
Source: Microb Genom. 2016 Dec 12;2(12):e000102. doi: 10.1099/mgen.0.000102 (PMC5359410; doi:10.1099/mgen.0.000102)
Supplement: Supplementary File 1 [file mgen-02-102-s001.pdf]

IDENTIFICATION OF *KLEBSIELLA* CAPSULE SYNTHESIS LOCI FROM GENOME DATA: Supplementary Materials

Supplementary Methods

Justification of approach

We focus on characterisation of K-loci comprising novel K-locus structures, i.e. identified on the basis of gene content rather than nucleotide diversity within genes. We assume that in most cases differences in gene repertoire are likely to be associated with differences in capsule phenotype as is the case for the majority of the serologically defined K-type reference strains (Pan *et al.*, 2015). The one known exception is the locus encoding K37, which is distinguished from that encoding K22 only by point mutation, although this too can be considered a difference in gene content since the point mutation results in truncation of an acetyltransferase gene encoded within the K-locus (Pan *et al.*, 2015). Most importantly for molecular surveillance purposes and evolutionary investigations, we assume that K-loci which differ only by nucleotide divergence (i.e. they contain the same complement of protein-coding genes with some nucleotide sequence variation within or between these genes) can be considered evolutionarily closely related, whereas those that differ in terms of gene content are more distantly related. An alternative approach would be to cluster full-length K-locus nucleotide sequences (Follador *et al.*, 2016). However, our gene-based approach has the advantage of grouping together K-loci that only differ due to assembly gaps, low-level nucleotide divergence or insertion sequence (IS) insertions, all of which commonly occur in K-loci (Wyres *et al.*, 2015).

Initial assignment of genomes to K-loci

We used BLASTn to screen genomes for the presence of coding sequences (CDS) from known K-loci as described in **Methods**. We tolerated up to three missing genes to allow for sequence or assembly errors and the effects of IS insertions that may interrupt CDS. We speculated that there would be two distributions for the number of missing K-locus genes in each genome assembly: (i) the distribution due to assembly problems and IS insertions in genomes with a true match to the K-locus; and (ii) the distribution due to differences in gene content between the true match locus and each of the other K-loci. The threshold of three missing genes was determined as the point of crossover of these two distributions as shown in **Fig. S1**.

Kaptive, a tool for identification of K-loci in genome data

We extended our BLAST-based K-locus typng approach and implemented the extended procedure in *Kaptive*, a freely available command-line tool that enables rapid identification of capsule loci from genome assemblies.

Given a query genome and a database of reference K-loci, *Kaptive* uses nucleotide alignments (Camacho *et al.*, 2009) to identify the closest matching K-locus in the database. It uses BLASTn to search for the entire length of each reference K-locus, and reports the best-match based on alignment coverage (and identity, in case of coverage tie). The region of the query assembly which matches the best-match K-locus is extracted. This region may

be on a single contig or split across multiple contigs. Next *Kaptive* performs a tBLASTn search for all K-locus genes (where predicted amino acid sequences are passed as the BLAST queries). The results for genes from the best-match locus (expected) are reported separately to those from other loci (unexpected). Furthermore, positive matches are partitioned into those identified within or outside the K-locus region extracted from the genome assembly. Overlapping matches e.g. for homologous genes are filtered such that only one match is reported for any given query assembly region. In such cases, expected gene matches are prioritised over unexpected matches. The analysis procedure is summarised in **Fig. 7** in the main text and described in detail below in the info-graphic below.

Kaptive does not assign new types to novel K-loci but rather attempts to provide enough information for users to determine whether putative novel K-loci are sufficiently distinct to warrant further investigation (as opposed to inadequate assembly coverage to identify the K-locus).

Kaptive requires two inputs: a database of reference K-loci (such as the *Klebsiella* databases described in this work and distributed with *Kaptive*) and one or more assembled genomes to analyse. The reference K-loci are provided in a multi-record GenBank file, where each record holds a K-locus reference with a nucleotide sequence and coding sequence annotations. The assembled genomes are provided in FASTA format (one file per genome, each containing one FASTA entry per assembled contig). High-quality assemblies (i.e. assemblies with long, unbroken contigs) allow for more informative results however, *Kaptive* is designed to work with low-quality fragmented assemblies as well.

Kaptive produces two types of output: tabulated K-locus results and sequences for the K-loci identified. The table summarises the K-locus analysis for each assembly: the best-match reference K-locus, which K-locus genes are present or absent, and possible problems with the match. Problems include; the K-locus region in the assembly is fragmented, an expected gene is missing from the K-locus region of the assembly, an unexpected gene is present in the K-locus region of the assembly and one or more expected genes are present at low identity (default <95%). For each genome, a FASTA format sequence file is generated containing the K-locus sequence(s) identified in the assembly. Ideally, this will be a single contiguous sequence extracted from a single contig, however if the assembly is fragmented then the K-locus may be in multiple pieces. Further information and examples are provided with the program. The *Klebsiella* K-locus databases described in this study are distributed with *Kaptive* to facilitate *Klebsiella* K-locus typing, however *Kaptive* is also suitable for use with any appropriately formatted database of sequences.

Although we strongly advocate for full length K-locus analysis rather than single gene based analyses, we acknowledge that comparison of nucleotide sequence variation in common K-locus genes can provide useful information for outbreak investigations etc. For this reason, and to allow backwards compatibility of results, Kaptive will also perform a BLASTn search to identify the best matching *wzi* and *wzc* alleles as defined in the *Kp* BIGSdb hosted at http://bigsdb.pasteur.fr/klebsiella/klebsiella.html. These results are reported in the main output table.

Kaptive analysis procedure

Input files

Kaptive requires two inputs:

- A reference K loci database in a single Genbank file. Each record holds a known K locus type with a nucleotide sequence and coding sequence annotations.
- One or more assembled genomes requiring K locus analysis. These are preferably high quality assemblies where the K locus sequence has assembled into a single contig.

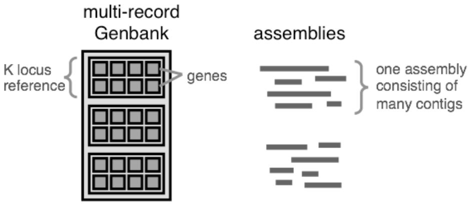

Kaptive analyses each assembly independently. The following steps 1-6 illustrate the process for a single input assembly.

1. Nucleotide BLAST

Kaptive conducts a nucleotide screen using BLASTn, with the assembly as the BLAST database and the K locus reference sequence as the BLAST query. A search is independently conducted using each K locus reference sequence.

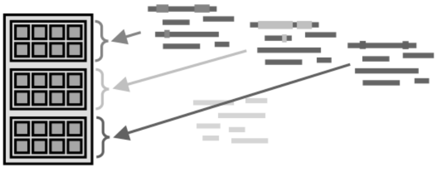

2. Choose best match

Kaptive chooses the best K locus reference based on BLAST coverage – i.e. the K locus reference for which the largest proportion aligns to the assembly. Coverage is used instead of alignment identity because the capsule phenotype is highly dependent on which genes are present and less dependent on variation within those genes.

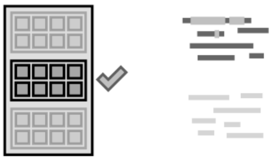

3. Get K locus region of assembly

In an ideal case, the best K locus match will consist of a single complete BLAST alignment to one contig in the assembly. However, the K locus region of the assembly may be broken into pieces or have unaligned parts, and there may be spurious alignments elsewhere in the assembly. *Kaptive* simplifies the results by discarding redundant alignments, merging neighbouring alignments and filtering out excessively small alignments. At the end of this process, the program will save the K locus region of the assembly (hopefully one contiguous piece but possibly multiple pieces) to a FASTA file.

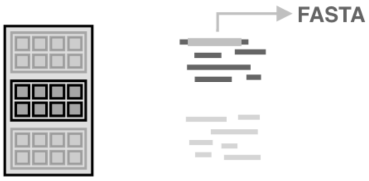

4. Gene BLAST

Kaptive now conducts a gene screen for all K locus genes using tBLASTn, with the assembly as the BLAST database and predicted proteins from all reference K loci as queries. Many of the queries may be very similar, as different reference K loci will contain similar alleles e.g. for the common genes, so many redundant BLAST alignments will be generated.

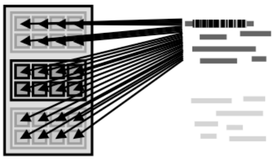

5. Filter overlapping alignments

Kaptive removes alignment redundancy by discarding overlapping tBLASTn alignments. An alignment is preferentially kept based on its identity, whether it is for an expected gene (i.e. a gene in the best matching K locus) and whether it was found in the K locus part of the assembly.

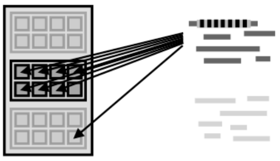

6. Summarise results

Finally, *Kaptive* uses the gathered information to produce a summary output to the user. This is written to a table and optionally displayed on the console as well. The table contains one row per assembly which has the following information: which K locus reference is the best match; coverage, identity and length discrepancy of the match; gene alignments and their identities; whether any expected genes are missing; and character codes for potential match problems.

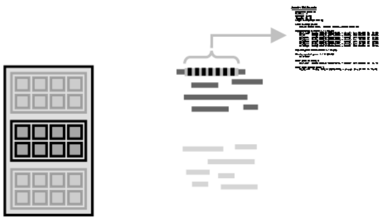

Repeat with next assembly

The same steps are then carried out on the remaining assemblies. Multiple copies of the program can be run simultaneously, all of which can add their results to the same output table. This allows large numbers of analyses to be completed in parallel.

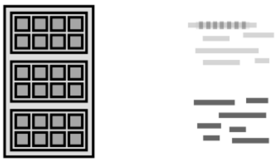

Sample *Kaptive* output table

Summary of potential problems with the match:
? = the K locus was not found in a single contiguous piece
+ = one or more additional genes were found in the K locus
- = one or more expected genes were missing from the K locus
* = one or more expected genes have low amino acid identity

Name of the input assembly, from the filename.

Name of the reference K locus with the best (highest coverage) match to the assembly.

Coverage and identity of the nucleotide match to the reference K locus.

Length discrepancy between the reference and assembled K loci (only applies when the assembly has a single contiguous K locus).

Number, gene names and protein identities of expected genes (genes in the reference K locus) found in the protein BLAST of the assembly.

Names of any expected genes which were not found in the protein BLAST.

Number, gene names and protein identities of unexpected genes (genes not in the reference K locus) found in the K locus part of the assembly.

Number, gene names and protein identities of expected genes found in the assembly but not in the K locus part.

Number, gene names and protein identities of unexpected genes found in the assembly but not in the K locus part.

Assembly	Best match locus	Problems	Coverage	Identity	Length discrepancy	Expected genes in locus	Expected genes in locus, details	Missing expected genes	Other genes in locus	Other genes in locus, details	Expected genes outside locus	Expected genes outside locus, details	Other genes outside locus	Other genes outside locus, details
sample_1	KL1		100.0%	99.9%	-2 bp	4 / 4 (100%)	KL1_abc,100.0%; KL1_bcd,99.4%; KL1_cde,99.7%; KL1_def,100.0%		0		0		1	KL17_qrs, 79.4%
sample_2	KL9	*	99.8%	95.3%	+97 bp	4 / 4 (100%)	KL9_abc,96.2%; KL9_bcd,98.1%; KL9_cde,92.9%; KL9_def,99.2%		0		0		1	KL19_qrs, 74.2%
sample_3	KL5	+	100.0%	97.4%	+3343 bp	4 / 4 (100%)	KL5_abc,97.9%; KL5_bcd,97.6%; KL5_cde,99.8%; KL5_def,96.1%		3	KL14_xyz,98.8%	0		1	KL17_qrs, 76.9%
sample_4	KL3	?	99.2%	95.1%	n/a	4 / 4 (100%)	KL5_abc,97.9%; KL5_bcd,97.6%; KL5_cde,99.8%; KL5_def,96.1%		0		0		1	KL16_qrs, 78.2%
sample_5	KL2	?-*	77.9%	85.8%	n/a	2/4 (75%)	KL3_abc,81.1%; KL3_def,77.5%	KL3_bde; KL3_cd	0		0		4	KL4_lmn, 81.3%; KL7_ijk,77.7%

Sample_1: very strong match

This is a case where our assembly very closely matches a known K locus. There are no characters in the 'Problems' column, the coverage and identity are both high, the length discrepancy is low, and all expected genes were found with high identity. A couple of other low-identity K locus gene alignments were elsewhere in the assembly, but that's not a concern; it is normal for a genome to contain homologous of genes found in some known K loci (see text below). Overall, this is a confident match for KL1.

Sample_2: more distant match

This sample matches the KL9 locus sequence, but not as closely as our previous case. The * character indicates that one or more expected genes fell below the identity threshold (default 95%). In the 'Expected genes in K locus, details' column, we can see that it is the K9_cde gene in this case. Our sample probably still has a KL9 locus, but it has diverged somewhat from the KL9 reference, possibly due to mutation and/or recombination.

Sample_3: acquired gene

This sample has a good match to the KL5 reference, with high nucleotide coverage and identity and high amino acid identities for the expected genes. However, the K locus also contains additional genes similar to those in KL14. This has also resulted in a large nucleotide length discrepancy.

Sample_4: broken assembly

This case has a '?' in the 'Problems' column, indicating that the K locus region of the assembly was not found in a single contiguous piece. This is likely because the assembly for this sample is fragmented and the K locus is split across multiple contigs. Fragmented assemblies can result from non-biological reasons, such as poor sequencing quality, or biological reasons, such as insertion sequences. So while the sample appears to strongly match KL3, we must view fragmented results with greater uncertainty.

Sample_5: poor match

This sample did not match any of the reference K loci well – even the best match has low coverage, low identity and missing genes. It may represent a novel K locus that requires closer examination outside of *Kaptive*.

Supplementary Results

K-locus reference databases

Where possible K-locus sequences were included at their full length, from the start of *galF* to the end of *ugd*. Where a previously published sequence did not span the full length of the locus or contained an IS, we substituted the complete, IS-free K-locus sequence from a genome in our collection if available (39 of 51). Where no naturally occurring IS-free variants were available, we manually generated an IS-free synthetic sequence (**Table S2**). IS-free sequences are included in a primary K-locus reference database, while all available IS or deletion variant K-locus references are included in an accompanying variant database, both available at https://github.com/katholt/Kaptive.

Application of Kaptive to the K. pneumoniae sensu lato genome collection

We ran *Kaptive* on all 2503 *K. pneumoniae* genomes in our collection. Here we summarise the results for the complete collection and explore in further detail the results for the 274 genomes from the global diversity collection described in (Holt *et al.*, 2015), which we believe is a fair representation of the *K. pneumoniae* population.

Kaptive will always return a 'best-match' K-locus plus a range of additional information to help the user decide if the best match is a good match (see above). In our genome collection, nucleotide coverage and identity of K-locus regions compared to the best match K-locus varied from 43.1% to 100% and 74.9 to 100% respectively. However, the distributions were highly skewed; 2300 of 2503 coverage results (91.8%) were ≥99% and 2387 results (95.4%) were ≥95% (median = 100%). Similarly 1926 identity results (76.9%) were ≥99% and 2391 results (95.5%) were ≥95% (median = 99.85%). Among genomes that had previously been assigned to K-loci (excluding deletion variants) by the gene screening approach these values were; coverage 71.6% to 100% and identity 90.1% to 100%.

The percentage of expected genes that were identified within the K-locus region of each assembly ranged from 18.8% to 100% (median = 100%, 2258 of 2508 (90.2%) results ≥90%). The number of unexpected genes identified within the K-locus region of each assembly ranged from 0 to 11 (median = 0, 2463 of 2503 results (98.4%) ≤ 1). Among the global diversity collection the percentage of expected genes identified within the K-locus region ranged from 33.3% to 100% and the number of unexpected genes within the locus ranged from 0 to 3 for 273 of 274 genomes and 11 for the remaining genome. The latter case represented an example of very low depth sequence read contamination that had passed our quality control criteria but had been identified and excluded in the previous analyses. The best-match locus identified by *Kaptive* was that of the true genome, while the additional 11 genes corresponded to those in the K-locus of the contaminant. There were 39 other genomes for which unexpected genes were identified in the K-locus region. In all but one case the unexpected genes were either homologues of genes that were expected but were not successfully identified or tBLASTn matches to *ugd*. The former occurs when the test genome contains a divergent version of the expected sequence, which does not meet the identity and/or coverage threshold cut-off and is more closely related to the homologous sequence encoded in a different K-locus reference. The latter occurs when the best match reference locus is incomplete, containing only a partial *ugd* sequence that was

consequently not annotated. In such a case *Kaptive* does not include *ugd* in the expected list, and thus reports only 'unexpected' matches.

A total of 1763 K-loci in our complete collection were present in their respective genome assemblies in single contiguous regions (70.4% genomes). Among the global diversity collection the K-locus was identified as a single contiguous sequence from 173 of 274 genomes (63.1%) and absolute length discrepancies to the best-match locus ranged from -615bp to +32bp for 171 of 173 genomes. For the remaining two genomes the discrepancies were +1050bp and +1095bp. Further investigation showed that these discrepancies corresponded to the acquisition of IS*903* and IS*630* elements in the K-loci of the test genomes, which were otherwise highly similar to those of the best-match references (98.53% nucleotide identity, 100% coverage to KL2 and 99.97% identity, 100% coverage to KL15, respectively).

Among the global diversity collection there were no cases were expected genes were identified outside of the K-locus region. However, in all cases there were between one and ten unexpected genes identified outside of the K-locus region. This is not surprising given that some K-locus assembly and synthesis genes share similarity to those associated with the synthesis of other surface antigens e.g. lipopolysaccharide (LPS), for which the genes are located in other regions of the genome. Such genes include *rmlBADC, manBC* and *gmd*. In addition, the KL40 and KL50 reference sequences actually contain coding regions from the LPS locus, presumably resulting from a translocation event in the reference strain genome. The LPS locus is located just downstream from the K-locus in the *Klebsiella* genome and shows a much lower level of diversity. Furthermore, LPS loci and K-loci appear to be subject to frequent shuffling, generating many distinct combinations (Follador *et al.*, 2016). It is therefore neither surprising, nor of concern that matches to these proteins are identified outside the putative K-locus regions in many genomes (e.g. matches to KL40 coding sequences 3, 4 and 5, and KL50 coding sequence 10 were identified among 82, 83, 161 and 23 genomes, respectively). However, cases for which there are matches to several other unexpected genes should be treated cautiously.

Kaptive - What is a good match?

We suggest that users make their own decisions about what constitutes a 'good' match for their intended purpose. For the purposes of this work we did not attempt to systematically identify and characterise variants of the reference K-loci (i.e. IS, frame shift mutation or deletion variants), although several were identified - the reference annotations for which are provided alongside *Kaptive* in a variants database. Given these requirements and the findings above, we designated the following confidence levels for this study:

1) High confidence calls - K-locus present in a single contiguous sequence with ≥99% BLASTn coverage to the reference sequence and no more than three expected genes missing within the locus. Note where ≥99% of a locus is identified by BLASTn some genes can be 'missing' if the test genome contains a divergent form of the gene compared to the reference (default threshold is 80% tBLASTn), or if there is a frame shift mutation in the centre of the gene such that the length of each of the resultant chunks is below the coverage threshold (default is 90%).
2) Good confidence calls - K-locus either present in a single contiguous sequence OR K-locus present in multiple pieces with ≥95% BLASTn coverage to the reference

sequence, at most three expected genes unidentified and no more than one unexpected gene identified in the K-locus region

3) Low confidence calls - K-locus in multiple pieces with ≥90% BLASTn coverage to the reference sequence and no more than two unexpected genes in the K-locus region
4) No confidence calls - <90% BLASTn coverage to the reference sequence and/or more than three expected genes missing form the K-locus region and/or more than two unexpected genes in the K-locus region.

***Kaptive* call rates**

We applied the call confidence criteria to the *Kaptive* results for our collection of 2503 *Klebsiella* genomes. We obtained 1715 (68.5%) high confidence calls, 610 (24.3%) good confidence calls and 87 (3.5%) low confidence calls. A small proportion (3.6%) of genomes could not be confidently called by *Kaptive*. These included many of those for which we were unable to assign a K-locus by the initial gene screening method (n = 62) and six genomes that were known to harbour K-locus deletion variants.

Supplementary Figures

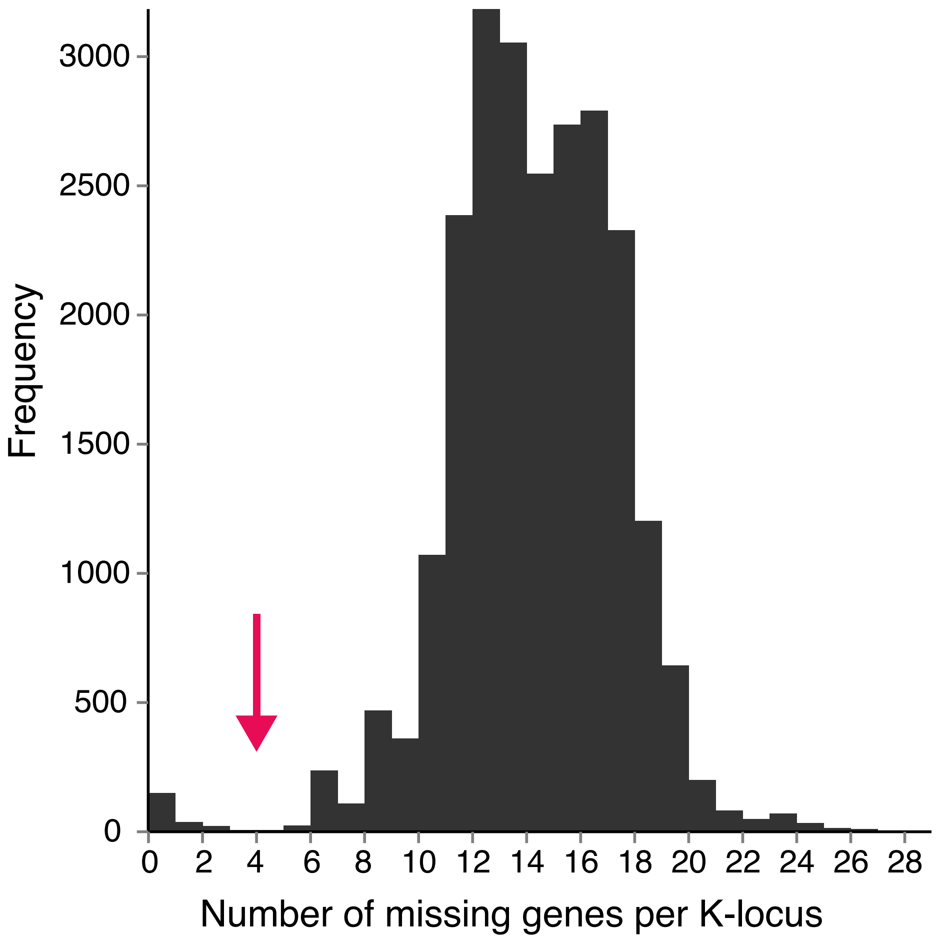

Figure S1: Distribution of missing genes per genome per K locus

Gene presence was determined by BLASTn search (minimum identity 50%, minimum coverage 80%). Data shown are for 274 genomes sequenced as part of a global diversity study by Holt *et al.* (Holt *et al.*, 2015). These genomes were screened for 86 distinct K loci published prior to 2016 (77 K-type reference K loci plus nine others). The red arrow indicates the point of cross-over of the two distributions.

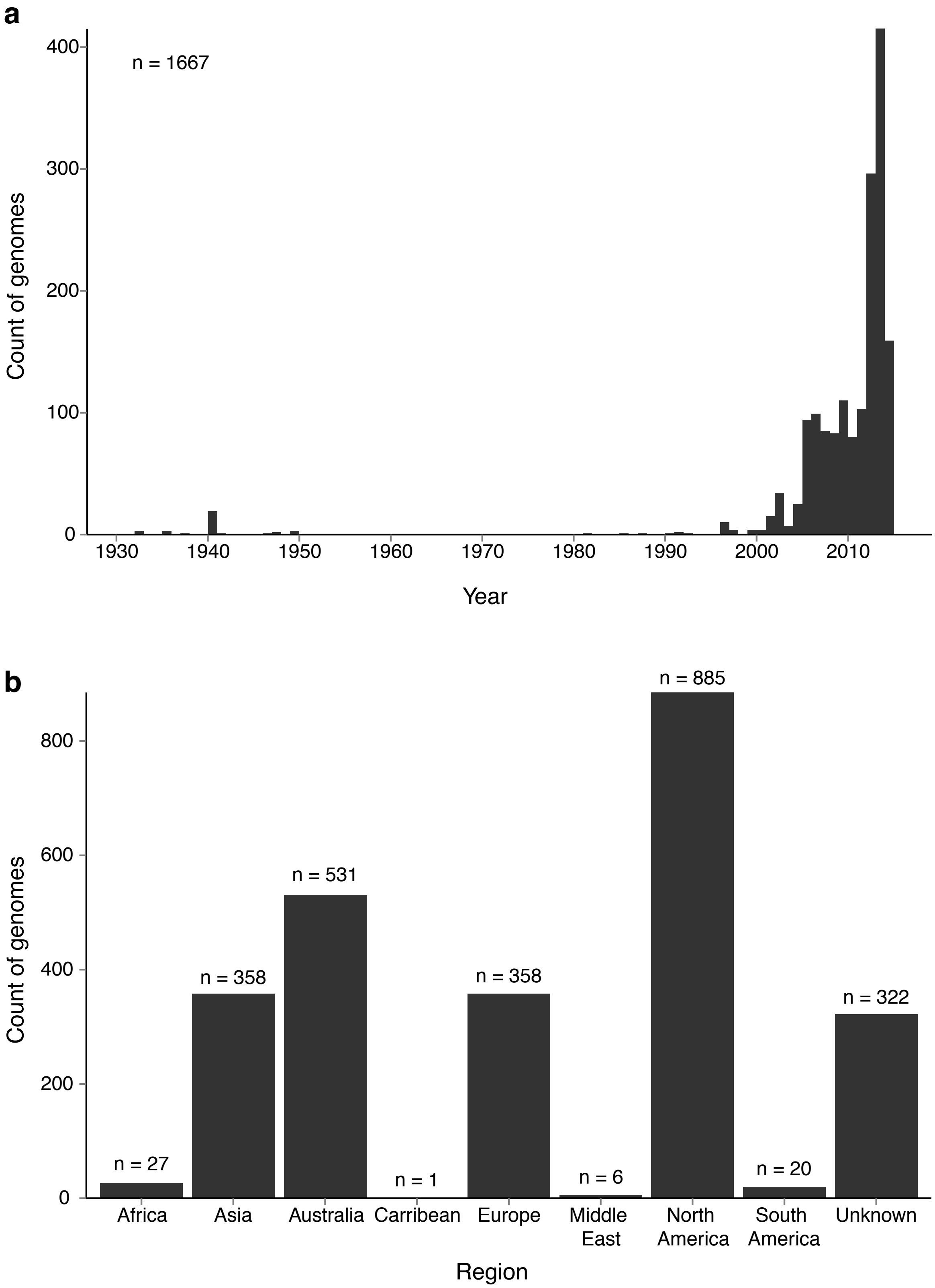

Figure S2: Distribution of *Klebsiella* genomes (a) by year of isolate collection and (b) by region of isolate collection, if known.

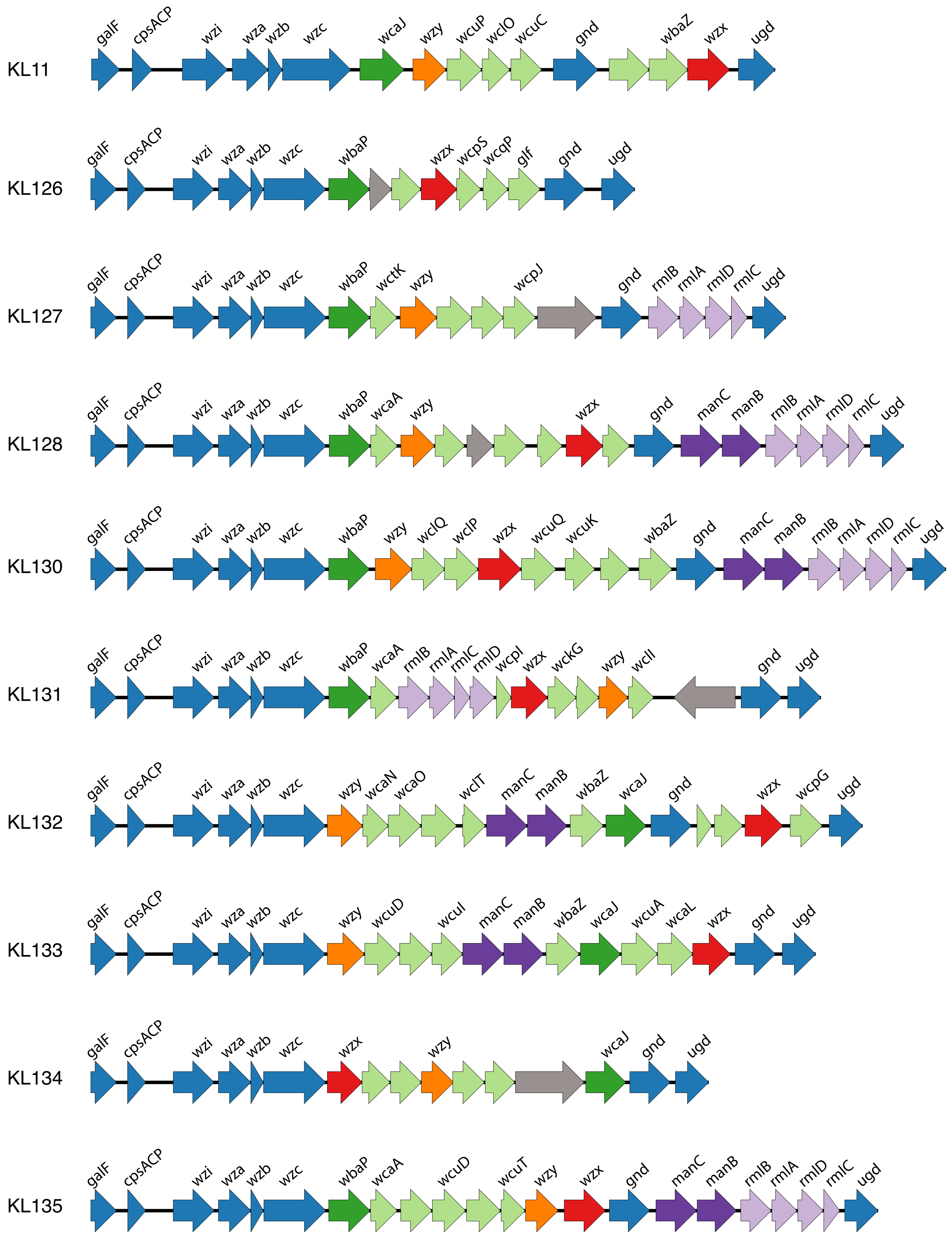
KL11
galF cpsACP wzi wza wzb wzc wcaJ wzy wcuP wclO wcuC gnd wbaZ wzx ugd
KL126
galF cpsACP wzi wza wzb wzc wbaP wzx wcpS wcqP glf gnd ugd
KL127
galF cpsACP wzi wza wzb wzc wbaP wctK wzy wcpJ gnd rmlB rmlA rmlD rmlC ugd
KL128
galF cpsACP wzi wza wzb wzc wbaP wcaA wzy wzx gnd manC manB rmlB rmlA rmlD rmlC ugd
KL130
galF cpsACP wzi wza wzb wzc wbaP wzy wclQ wclP wzx wcuQ wcuK wbaZ gnd manC manB rmlB rmlA rmlD rmlC ugd
KL131
galF cpsACP wzi wza wzb wzc wbaP wcaA rmlB rmlA rmlC rmlD wcpI wzx wckG wzy wclI gnd ugd
KL132
galF cpsACP wzi wza wzb wzc wzy wcaN wcaO wclT manC manB wbaZ wcaJ gnd wzx wcpG ugd
KL133
galF cpsACP wzi wza wzb wzc wzy wcuD wcuI manC manB wbaZ wcaJ wcuA wcaL wzx gnd ugd
KL134
galF cpsACP wzi wza wzb wzc wzx wzy wcaJ gnd ugd
KL135
galF cpsACP wzi wza wzb wzc wbaP wcaA wcuD wcuT wzy wzx gnd manC manB rmlB rmlA rmlD rmlC ugd

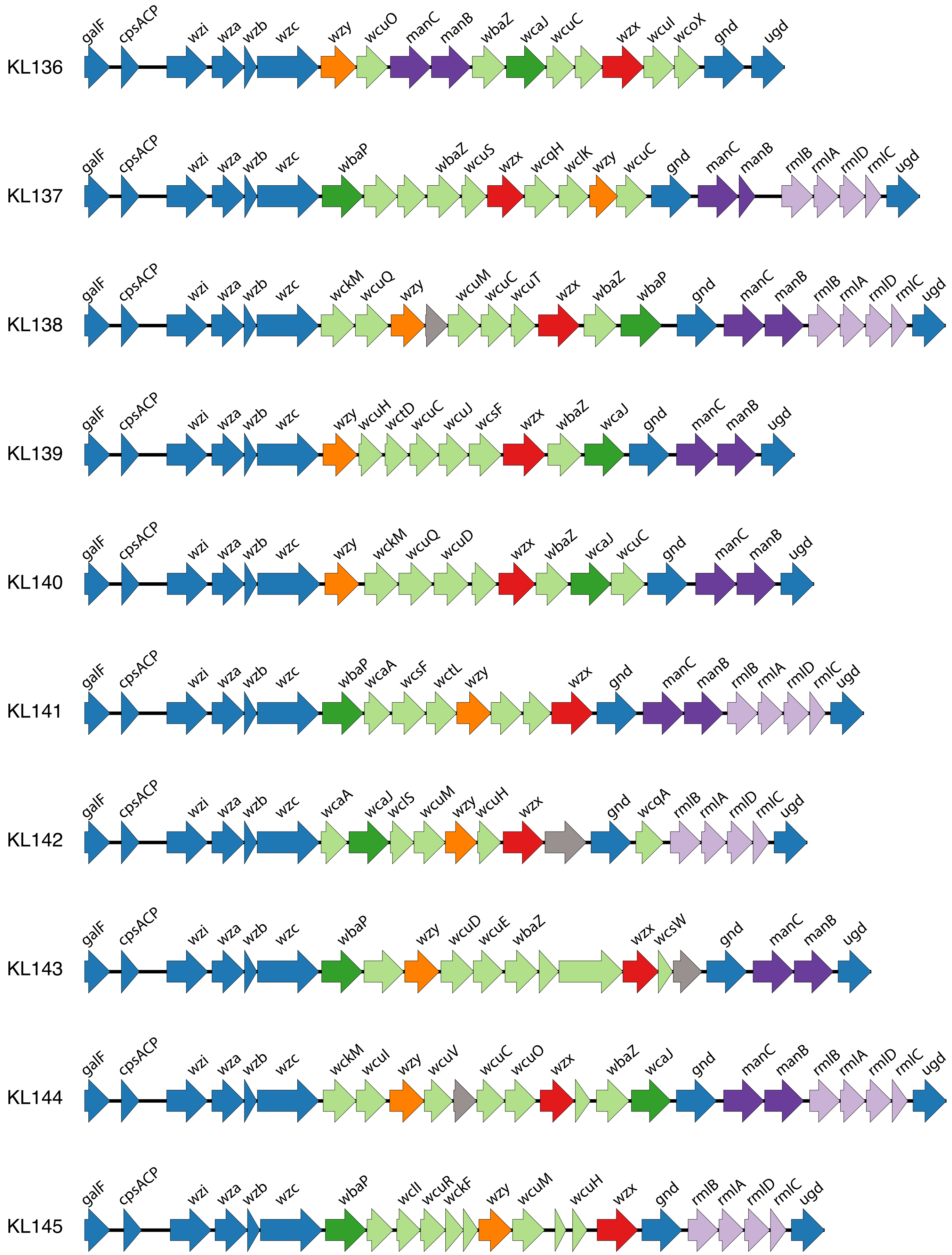
KL136
galF cpsACP wzi wza wzb wzc wzy wcuO manC manB wbaZ wcaJ wcuC wzx wcuI wcoX gnd ugd
KL137
galF cpsACP wzi wza wzb wzc wbaP wbaZ wcuS wzx wcqH wclK wzy wcuC gnd manC manB rmlB rmlA rmlD rmlC ugd
KL138
galF cpsACP wzi wza wzb wzc wckM wcuQ wzy wcuM wcuC wcuT wzx wbaZ wbaP gnd manC manB rmlB rmlA rmlD rmlC ugd
KL139
galF cpsACP wzi wza wzb wzc wzy wcuH wctD wcuC wcuJ wcsF wzx wbaZ wcaJ gnd manC manB ugd
KL140
galF cpsACP wzi wza wzb wzc wzy wckM wcuQ wcuD wzx wbaZ wcaJ wcuC gnd manC manB ugd
KL141
galF cpsACP wzi wza wzb wzc wbaP wcaA wcsF wctL wzy wzx gnd manC manB rmlB rmlA rmlD rmlC ugd
KL142
galF cpsACP wzi wza wzb wzc wcaA wcaJ wclS wcuM wzy wcuH wzx gnd wcqA rmlB rmlA rmlD rmlC ugd
KL143
galF cpsACP wzi wza wzb wzc wbaP wzy wcuD wcuE wbaZ wzx wcsW gnd manC manB ugd
KL144
galF cpsACP wzi wza wzb wzc wckM wcuI wzy wcuV wcuC wcuO wzx wbaZ wcaJ gnd manC manB rmlB rmlA rmlD rmlC ugd
KL145
galF cpsACP wzi wza wzb wzc wbaP wclI wcuR wckF wzy wcuM wcuH wzx gnd rmlB rmlA rmlD rmlC ugd

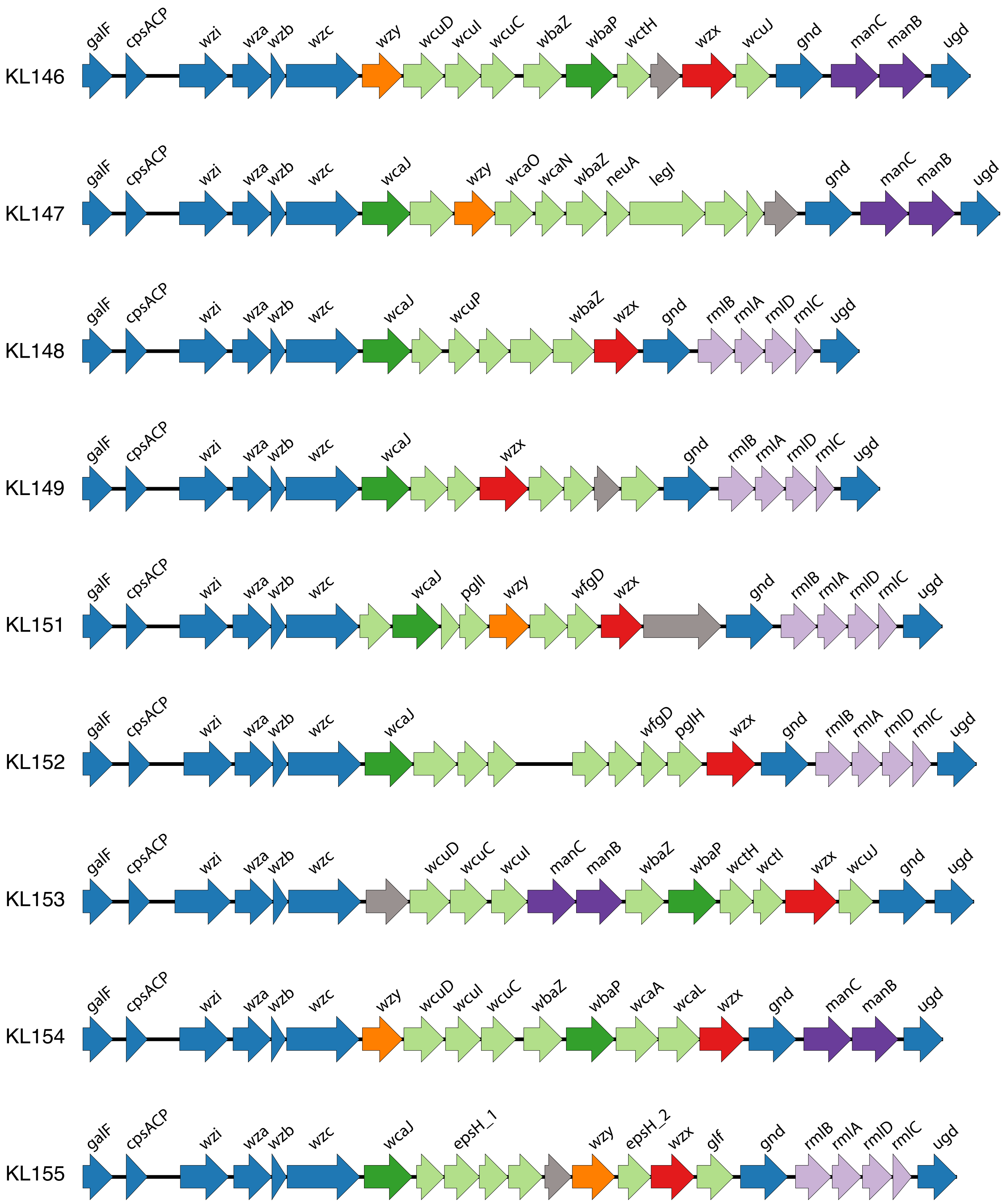
KL146
galF cpsACP wzi wza wzb wzc wzy wcuD wcuI wcuC wbaZ wbaP wctH wzx wcuJ gnd manC manB ugd
KL147
galF cpsACP wzi wza wzb wzc wcaJ wzy wcaO wcaN wbaZ neuA legI gnd manC manB ugd
KL148
galF cpsACP wzi wza wzb wzc wcaJ wcuP wbaZ wzx gnd rmlB rmlA rmlD rmlC ugd
KL149
galF cpsACP wzi wza wzb wzc wcaJ wzx gnd rmlB rmlA rmlD rmlC ugd
KL151
galF cpsACP wzi wza wzb wzc wcaJ pglI wzy wfgD wzx gnd rmlB rmlA rmlD rmlC ugd
KL152
galF cpsACP wzi wza wzb wzc wcaJ wfgD pglH wzx gnd rmlB rmlA rmlD rmlC ugd
KL153
galF cpsACP wzi wza wzb wzc wcuD wcuC wcuI manC manB wbaZ wbaP wctH wctI wzx wcuJ gnd ugd
KL154
galF cpsACP wzi wza wzb wzc wzy wcuD wcuI wcuC wbaZ wbaP wcaA wcaL wzx gnd manC manB ugd
KL155
galF cpsACP wzi wza wzb wzc wcaJ epsH_1 wzy epsH_2 wzx glf gnd rmlB rmlA rmlD rmlC ugd

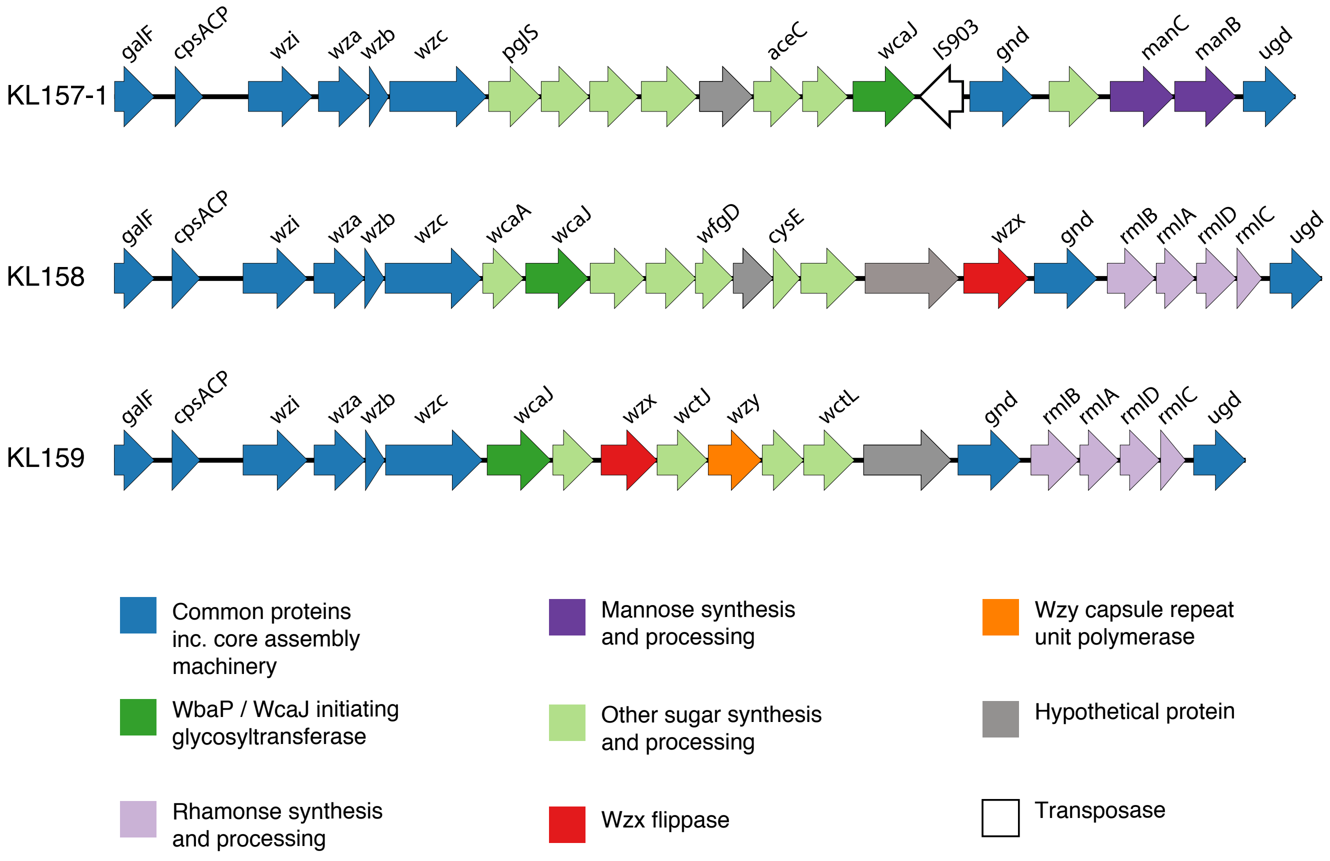

Figure S3: Structures of *Klebsiella* K loci newly identified in this study
Coding sequences are represented by arrows coloured by predicted protein products as indicated. A complete K locus, which we argue represents the true KL11 structure (See main text) was identified in this work and is shown here.

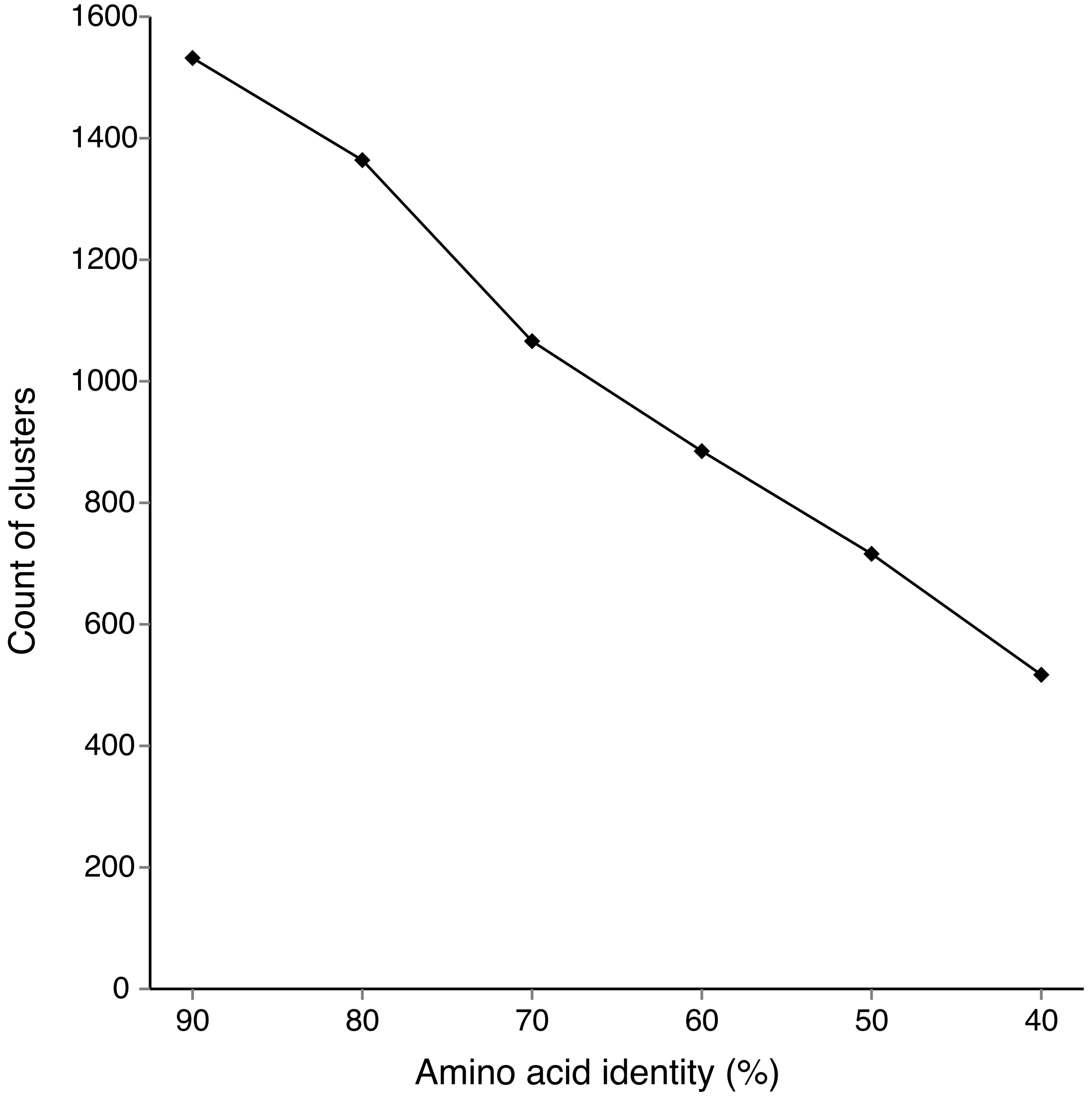

Figure S4: Count of predicted K locus amino acid sequence clusters at varying identity levels

Amino acid sequences were predicted for coding regions in all 134 curated K locus references and clustered with CD-HIT (Fu *et al.*, 2012; Li & Godzik, 2006).

Supplementary Tables

Table S1: ***K. pneumoniae*** **genome data analysed in this study**
Accession numbers, K-locus designations and summarised *Kaptive* typing results are provided. See separate Excel sheet.

Table S2: ***Klebsiella*** **K-locus references, accession numbers and isolate names**
See separate Excel sheet.

Table S3: ***Kaptive*****,** ***wzi, wzc*** **and serological typing results for 86** ***K. pneumoniae*** **genomes for which serological typing information were available**
See separate Excel sheet.

Table S4: ***Klebsiella oxytoca*** **genomes included in this study**

Accession	Reference	KL-type
CP003218	(Shin *et al.*, 2012)	KL152
NC_018106	(Liao *et al.*, 2012)	KL145
SRR1508828	(Stoesser *et al.*, 2014)	Unknown
SRR1508867	(Stoesser *et al.*, 2014)	KL74
SRR1508868	(Stoesser *et al.*, 2014)	KL74
SRR1508869	(Stoesser *et al.*, 2014)	KL74
SRR1508870	(Stoesser *et al.*, 2014)	Unknown
SRR1508871	(Stoesser *et al.*, 2014)	KL74
SRR1508878	(Stoesser *et al.*, 2014)	KL66
SRR1508880	(Stoesser *et al.*, 2014)	KL152

Table S5: Jaccard similarity scores for 115 K-locus protein clusters for which the associated genes were present in at least three K-loci
See separate Excel sheet.

References

Camacho, C., Coulouris, G., Avagyan, V., Ma, N., Papadopoulos, J., Bealer, K. & Madden, T. L. (2009). BLAST+: architecture and applications. *BMC Bioinformatics* **10**, 421.

Follador, R., Heinz, E., Wyres, K. L., Ellington, M. J., Kowarik, M., Holt, K. E. & Thomson, N. R. (2016). The diversity of *Klebsiella pneumoniae* surface polysaccharides. *MGen* **2**.

Fu, L., Niu, B., Zhu, Z., Wu, S. & Li, W. (2012). CD-HIT: Accelerated for clustering the next-generation sequencing data. *Bioinformatics* **28**, 3150–3152.

Holt, K. E., Wertheim, H., Zadoks, R. N., Baker, S., Whitehouse, C. A., Dance, D., Jenney, A., Connor, T. R., Hsu, L. Y. & other authors. (2015). Genomic analysis of diversity,

population structure, virulence, and antimicrobial resistance in *Klebsiella pneumoniae*, an urgent threat to public health. *Proc Natl Acad Sci U S A* **112**, E3574-81.

Li, W. & Godzik, A. (2006). CD-Hit: A fast program for clustering and comparing large sets of protein or nucleotide sequences. *Bioinformatics* **22**, 1658–1659.

Liao, T. L., Lin, A. C., Chen, E., Huang, T. W., Liu, Y. M., Chang, Y. H., Lai, J. F., Lauderdale, T. L., Wang, J. T. & other authors. **(2012).** Complete genome sequence of *Klebsiella oxytoca* E718, a New Delhi metallo-beta-lactamase-1-producing nosocomial strain. *J Bacteriol* **194**, 5454–5454.

Pan, Y.-J., Lin, T.-L., Chen, C.-T., Chen, Y.-Y., Hsieh, P.-F., Hsu, C.-R., Wu, M.-C. & Wang, J.-T. (2015). Genetic analysis of capsular polysaccharide synthesis gene clusters in 79 capsular types of *Klebsiella* spp. *Nat Sci Rep* **5**, 15573.

Shin, S. H., Kim, S., Kim, J. Y., Lee, S., Um, Y., Oh, M. K., Kim, Y. R., Lee, J. & Yang, K. S. (2012). Complete genome sequence of Klebsiella oxytoca KCTC 1686, used in production of 2,3-butanediol. *J Bacteriol* **194**, 2371–2372.

Stoesser, N., Giess, A., Batty, E. M., Sheppard, A. E., Walker, A. S., Wilson, D. J., Didelot, X., Bashir, A., Sebra, R. & other authors. **(2014).** Genome sequencing of an extended series of NDM-producing *Klebsiella pneumoniae* isolates from neonatal infections in a Nepali hospital characterizes the extent of community- versus hospital- associated transmission in an endemic setting. *Antimicrob Agents Chemother* **58**, 7347–7357.

Wyres, K. L., Gorrie, C., Edwards, D. J., Wertheim, H. F. L., Hsu, L. Y., Van Kinh, N., Zadoks, R., Baker, S. & Holt, K. E. (2015). Extensive capsule locus variation and large-scale genomic recombination within the *Klebsiella pneumoniae* clonal group 258. *Genome Biol Evol* **7**, 1267–1279.
